# Ectomycorrhizal fungi decompose soil organic matter using oxidative mechanisms adapted from saprotrophic ancestors

**DOI:** 10.1111/nph.13722

**Published:** 2015-11-03

**Authors:** Firoz Shah, César Nicolás, Johan Bentzer, Magnus Ellström, Mark Smits, Francois Rineau, Björn Canbäck, Dimitrios Floudas, Robert Carleer, Gerald Lackner, Jana Braesel, Dirk Hoffmeister, Bernard Henrissat, Dag Ahrén, Tomas Johansson, David S. Hibbett, Francis Martin, Per Persson, Anders Tunlid

**Affiliations:** ^1^ Department of BiologyMicrobial Ecology GroupLund UniversityEcology BuildingSE‐223 62LundSweden; ^2^ Centre for Environmental SciencesHasselt UniversityBuilding DAgoralaan3590DiepenbeekLimburgBelgium; ^3^ Biology DepartmentLasry Center for BioscienceClark University950 Main StreetWorcesterMA01610‐1477USA; ^4^ Department of Pharmaceutical Microbiology at the Hans Knöll InstituteFriedrich‐Schiller‐UniversitätBeutenbergstrasse 11a07745JenaGermany; ^5^ Centre National de la Recherche Scientifique (CNRS)UMR7257Université Aix‐MarseilleMarseille13288France; ^6^ Department of Biological SciencesKing Abdulaziz UniversityJeddahSaudi Arabia; ^7^ Bioinformatics Infrastructures for Life Sciences (BILS)Department of BiologyLund UniversityEcology BuildingSE‐223 62LundSweden; ^8^ Institut de la Recherche Agronomique (INRA)Laboratory of Excellence ARBREUMR INRA‐Université de Lorraine ‘Interactions Arbres/Micro‐organismes’INRA‐Nancy54280ChampenouxFrance; ^9^ Centre for Environmental and Climate Research (CEC)Lund UniversityEcology BuildingSE‐223 62LundSweden

**Keywords:** decomposition, ectomycorrhizal fungi, evolution, soil organic matter, spectroscopy, transcriptome

## Abstract

- Ectomycorrhizal fungi are thought to have a key role in mobilizing organic nitrogen that is trapped in soil organic matter (SOM). However, the extent to which ectomycorrhizal fungi decompose SOM and the mechanism by which they do so remain unclear, considering that they have lost many genes encoding lignocellulose‐degrading enzymes that are present in their saprotrophic ancestors.
- Spectroscopic analyses and transcriptome profiling were used to examine the mechanisms by which five species of ectomycorrhizal fungi, representing at least four origins of symbiosis, decompose SOM extracted from forest soils.
- In the presence of glucose and when acquiring nitrogen, all species converted the organic matter in the SOM extract using oxidative mechanisms. The transcriptome expressed during oxidative decomposition has diverged over evolutionary time. Each species expressed a different set of transcripts encoding proteins associated with oxidation of lignocellulose by saprotrophic fungi. The decomposition ‘toolbox’ has diverged through differences in the regulation of orthologous genes, the formation of new genes by gene duplications, and the recruitment of genes from diverse but functionally similar enzyme families.
- The capacity to oxidize SOM appears to be common among ectomycorrhizal fungi. We propose that the ancestral decay mechanisms used primarily to obtain carbon have been adapted in symbiosis to scavenge nutrients instead.

## Introduction

A large part of the nitrogen (N) found in forest soils is present in organic forms, primarily as proteins and peptides but also as amino acids, amino sugars and heterocyclic molecules (Nannipieri & Eldor, 2009). During decomposition, these N molecules accumulate in a complex mixture of plant‐ and microbial‐derived molecules. This soil organic matter (SOM), operationally defined as humic substances, consists of relatively low‐molecular‐weight fragments of lignin, polysaccharides, polyphenols, lipids, peptidoglycan, peptides and other biomolecules (Simpson *et al*., 2007; Schmidt *et al*., 2011). The molecules associate with each other in supramolecular aggregates that are stabilized by hydrophobic interactions and hydrogen (H) bonding and by interactions with mineral particles (Kleber & Johnson, 2010; Kleber *et al*., 2015). To become available for plants, the organic N compounds must be released from the SOM. However, the microorganisms and the mechanisms involved in these processes are poorly characterized.

Filamentous saprotrophic fungi are thought to have a unique ability to degrade lignin and other phenolic compounds of forest SOM (Baldrian, 2008). On the basis of studies of wood‐decaying fungi, two major decomposition mechanisms have been characterized in detail: decomposition by white‐rot (WR) fungi and decomposition by brown‐rot (BR) fungi (Hatakka & Hammel, 2010). The ligninolytic system of WR fungi depends on extracellular oxidative enzymes, particularly class II peroxidases (class II PODs). These fungi also secrete various glycoside hydrolases (GHs) that break down crystalline cellulose. BR fungi lack most of the oxidative enzymes and cellulase systems of WR fungi (Floudas *et al*., 2012) and instead decompose lignocellulose by means of an initial nonenzymatic step: attack by reactive oxygen species, including hydroxyl radicals generated by the Fenton reaction (H_2_O_2_ + Fe^2+^ + H^+^ → H_2_O + Fe^3+^ + •OH) (Martinez *et al*., 2009; Hatakka & Hammel, 2010). In recent years, a number of new enzymes have been identified that are involved in the oxidative conversion of lignocellulose, such as lytic polysaccharide monooxygenases (LPMOs) (Quinlan *et al*., 2011), various oxidoreductases (Levasseur *et al*., 2013), and heme‐containing haloperoxidases, including chloroperoxidases and aromatic peroxygenases (Hofrichter *et al*., 2010). During fungal attack, these oxidases promote extended cleavage of plant cell wall components and generate the reactants needed for the Fenton reaction. In addition, secondary metabolites such as hydroquinones and variegatic acid have key roles in the reductions of Fe^3+^ and O_2_ in BR fungi (Eastwood *et al*., 2011; Korripally *et al*., 2013).

The other main functional group of fungi in northern forest soils comprises the ectomycorrhizal (ECM) symbionts. ECM fungi are biotrophs that obtain photosynthetic sugars from the host plant, which, in return, receives nutrients, including N and phosphorus, from the fungal partner (Smith & Read, 2008). Carbon (C) from the plant supports the growth of an extramatrical mycelium. This mycelium is present mainly in regions of the soil horizons that are rich in humic substances (Lindahl *et al*., 2007). To release the N present in such material, it can be expected that ECM fungi can disrupt organic‐matter‐N complexes. Indeed, studies using various model compounds such as cellulose and polyphenols have shown that ECM fungi have some decomposing capacity (Norkrans, 1950; Trojanowski *et al*., 1984). ECM fungi also produce extracellular enzymes such as cellulases, hemicellulases and polyphenoloxidases, which are thought to contribute to the degradation of components of plant litter (Read & Perez‐Moreno, 2003). However, ECM fungi express such enzymes at much lower levels than do saprotrophic fungi grown under identical conditions (Read & Perez‐Moreno, 2003).

Recent analyses of genome sequences further support the view that ECM fungi have a limited capacity to decompose complex organic material such as lignocellulose. These analyses have shown that ECM fungi have a smaller set of genes encoding enzymes that degrade plant cell walls than do their saprotrophic ancestors, including BR and WR wood decayers (Martin *et al*., 2008; Kohler *et al*., 2015). We previously demonstrated that the ECM fungus *Paxillus involutus* can nevertheless decompose lignocellulosic material in SOM extracts while assimilating organic N using a Fenton‐based oxidation mechanism similar to that of BR fungi (Rineau *et al*., 2012). The oxidation is triggered by the addition of glucose, which suggests that the mechanism can be regulated by the host C supply (Rineau *et al*., 2013). During the oxidative decomposition of the SOM extract, *P. involutus* expresses a large number of extracellular endo‐ and exopeptidases that are regulated in parallel with transporters and enzymes involved in the assimilation and metabolism of the released N (Shah *et al*., 2013). Other ECM taxa, including *Cortinarius* species, contain genes encoding class II PODs (Bödeker *et al*., 2009; Hatakka & Hammel, 2010). More recently, Bödeker *et al*. (2014) demonstrated that the transcription of *Cortinarius* class II PODs genes was correlated with high peroxidase activity in soils, supporting the hypothesis that *Cortinarius* species may play an important role in the decomposition of complex humic‐rich SOM in northern forest ecosystems.

Here, we examined the capacity of several ECM fungi to decompose biomolecules present in SOM and the mechanisms underlying this decomposition. In particular, we assessed the following: whether the capacity to decompose SOM has been retained by ECM fungi of different evolutionary origins and functional ecologies; whether the arrays of enzymes expressed upon SOM decomposition by ECM fungi are similar to those of related saprotrophic wood decomposers; and whether the decomposition mechanisms of ECM fungi have any common molecular signatures as a result of similar selection pressures.

## Materials and Methods

### Fungal species

**Figure 1:**
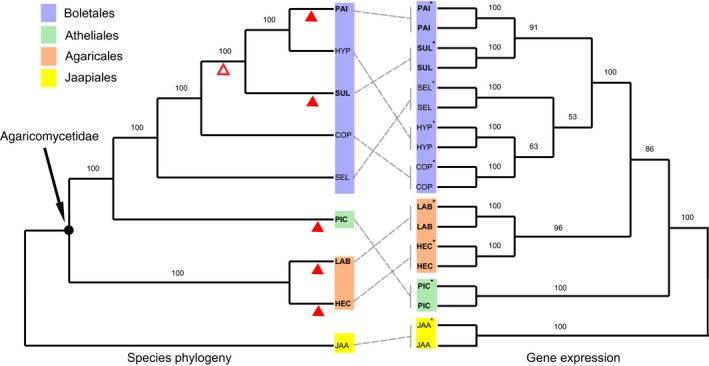
Species and gene expression phylogenies of analysed fungi. A maximum likelihood species tree (left) was reconstructed with sequences from 3148 putative 1: 1 orthologues from each of the nine analysed fungal species, with 1000 bootstrap replicates. The neighbour‐joining gene expression tree (right) was constructed with the expression levels of the orthologues upon fungal growth on soil organic matter extracts (asterisk) and mineral nutrient medium (MMN) (no asterisk), with 1000 bootstrap replicates. Both trees were rooted with *Jaapia argillacea* as an outgroup. The closed red triangles indicate the estimated origins of the ectomycorrhizal (ECM) fungi; the open red triangle indicates an alternative reconstruction with a single origin in the Boletales clade and at least one reversal to saprotrophy (Kohler *et al*., 2015). The last common ancestor of the Agaricomycetidae clade (indicated with an arrow) probably lived between 125 and 150 million yr ago (Floudas *et al*., 2012; Kohler *et al*., 2015). The designations in boldface letters indicate ECM fungi. COP,* Coniophora puteana*; HEC,* Hebeloma cylindrosporum*; HYP,* Hydnomerulius pinastri*; JAA,* Jaapia argillacea*; LAB,* Laccaria bicolor*; PAI,* Paxillus involutus*; PIC,* Piloderma croceum*; SEL,* Serpula lacrymans*; SUL,* Suillus luteus*.

We analysed five ECM species belonging to three basidiomycete orders (Boletales, Agaricales and Atheliales), selected based on the availability of published genome sequences. The five ECM species represent at least four independent evolutionary origins of symbiosis (Fig. 1; Supporting Information Table S1) and they are distant from each other as the last common ancestor of Agaricomycetidae probably lived between 125 and 150 million yr ago (Ma; Floudas *et al*., 2012; Kohler *et al*., 2015). The most densely sampled clade was the Boletales, with two ECM species (*Paxillus involutus* (Batsch) Fr. and *Suillus luteus* (L.) Roussel) that are nested with a paraphyletic assemblage of BR wood decayers (Kohler *et al*., 2015), of which three species (*Coniophora puteana* (Schumach.) P. Karst, *Hydnomerulius pinastri* (Fr.) Jarosch & Besl, and *Serpula lacrymans* (Wulfen) P. Karst.) were included in our study. The two examined ECM species of the Agaricales clade evolved independently from different saprotrophic ancestors: *Laccaria bicolor* (Maire) P.D. Orton may be derived from litter‐decomposing saprotrophs, whereas *Hebeloma cylindrosporum* Romagnesi is nested in a clade of WR wood decayers (Matheny *et al*., 2006; Kohler *et al*., 2015). Finally, *Piloderma croceum* J. Erikss. & Hjortstam is nested within the ecologically diverse Atheliales–Amylocorticales clade, which includes WR and BR saprotrophs as well as various biotrophs (Hibbett *et al*., 2014; Kohler *et al*., 2015). The wood‐decomposing fungus *Jaapia argillacea* Bres. (Jaapiales), which lacks ligninolytic class II PODs but encodes diverse enzymes acting on crystalline cellulose (Riley *et al*., 2014), was used as an outgroup. The sampled ECM fungi differ also in their ecology. The Boletales species are long‐distance‐exploration types with a rapidly growing extramatrical mycelium and a high capacity to decompose and mobilize organic N. By contrast, the ECM species from the other clades are short‐ and medium‐distance‐exploration types, which grow more slowly and have a limited ability to assimilate organic N (Agerer, 2001; Hobbie & Agerer, 2010).

### Culture conditions

The fungi were grown in Petri dishes on a layer of glass beads immersed in a minimum Melin–Norkrans (MMN) medium (2.5 g l^−1^ glucose, 500 mg l^−1^ KH_2_PO_4_, 200 mg l^−1^ NH_4_Cl, 150 mg l^−1^ MgSO_4_·7H_2_O, 25 mg l^−1^ NaCl, 50 mg l^−1^ CaCl_2_, 12 mg l^−1^ FeCl_3_·6H_2_O and 1 mg l^−1^ thiamine‐HCl; pH 4.0) (Rineau *et al*., 2012). After 9 d of incubation (18°C in the dark), the medium was replaced with MMN medium without N to induce an N‐deprived mycelium (Shah *et al*., 2013). After 24 h, the mycelium was washed in sterile water, and the SOM extract (10 ml) was added.

**Figure 2:**
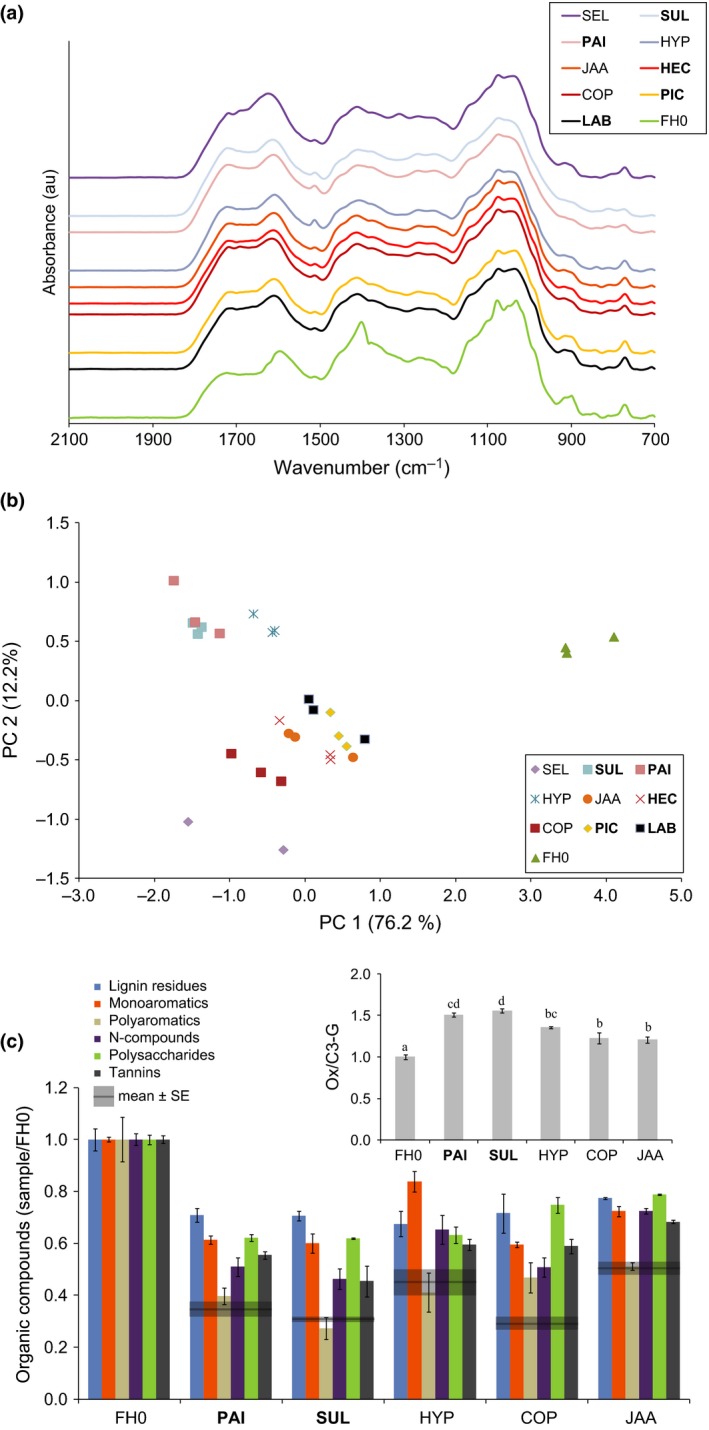
Decomposition of soil organic matter (SOM) extract. (a) Fourier transform infrared (FTIR) spectra of the SOM extract before (FH0, initial material) and after 7 d of incubation with various ectomycorrhizal (ECM) and saprophytic fungi (au, arbitrary units). All spectra have been normalized to the same total area over the wave number region displayed (*n* = 3). Spectral changes were observed in six regions ascribed to different vibrational modes: C–O and C–O–C stretching of carbohydrates (970–1150 cm^−1^); C–O stretching of phenols (1150–1250 cm^−1^); C–O stretching of esters (1300 cm^−1^); O–H bending, aliphatic C–H deformation or ammonium N–H bending (1350–1450 cm^−1^); C–C stretching of aromatic rings (1510 cm^−1^); and C=O stretching of carbonyl groups (1620–1800 cm^−1^). (b) Principal component analysis (PCA) scores plot of the FTIR spectra of the SOM extract before (FH0) and after 7 d incubation with the ECM fungi and saprotrophic fungi (*n* = 3). (c) Pyrolysis‐GC/MS results (shown as sums of the major groups of organic compounds). The data are corrected for the total organic C concentration and normalized to the nonincubated SOM extract (FH0) (mean ± SE;* n* = 3). The identified pyrolytic compounds are listed in Supporting Information Table S2. The inset shows the ratio of guaiacylacetone to *trans*‐propenylguaiacol (Ox/C3‐G) (grey bars), which is a marker of the degree of oxidation of guaiacyl lignin (Buurman *et al*., 2008). Bars with different lowercase letters are significantly different according to Tukey's test (*P* < 0.05). The values are normalized to the nonincubated samples (mean ± SE;* n* = 3). The species abbreviations are listed in the legend of Fig. 1.

SOM was extracted from the upper 10‐cm soil layer in a 61‐yr‐old Norway spruce (*Picea abies* (L.) H. Karst) stand growing in an N‐poor site in central Sweden (soil pH 5.0) (Table S2) using hot water (Davidson *et al*., 1987). Pyrolysis‐GC/MS (py‐GC/MS) (Fig. 2c) showed that the SOM extract contained the major classes of biomolecules that are present in intact SOM (Simpson *et al*., 2007). Particles were removed by filtration (0.2 μm), and low‐molecular‐weight metabolites were partly removed by ultrafiltration (cut‐off 1 kDa). The concentration of the SOM extract was adjusted, and the extract was supplemented with glucose to a final concentration similar to that in the MMN medium (Rineau *et al*., 2012). The fungi were grown in either the MMN medium or the SOM extract (three replicates each). The cultures were incubated for 7 d at 18°C in the dark. To test the capacity of the fungi to oxidize the organic matter in the absence of glucose, four of the Boletales species were grown on the SOM extract without glucose amendment.

### Chemical analyses

Total organic C concentration was measured with an organic C analyser (Shimadzu, Kyoto, Japan). Total N content was measured with the same apparatus equipped with a total nitrogen module (TNM‐1) detector. The glucose concentration was measured by means of a glucose assay (Sigma‐Aldrich, Seelze, Germany). Fourier transform infrared (FTIR) spectra were recorded on a Bruker IFS66 v/s spectrometer (Bruker Scientific Instruments, Billerica, MA, USA). Data were collected in diffuse reflectance mode. Each spectrum was the result of 1000 consecutive scans at a resolution of 4 cm^−1^. A Perkin‐Elmer TurboMass/Autosystem XL with a Frontier Lab Double Shot pyrolyser was used for py‐GC/MS (Perkin‐Elmer, Waltham, MA, USA). Pyrolysis data were acquired and processed with qcalibur 1.4 sr1 software (Thermo Finnigan, San Jose, CA, USA), and peaks were identified by comparison with published and stored data (National Institute of Standards and Technology (NIST) library). A standard series of SOM extracts with increasing concentrations of glucose (0.0–2.5 g l^−1^ glucose) was analysed to correct the contribution of glucose to each pyrolytic product. The iron‐reducing activity in the culture filtrates was analysed by means of a ferrozine assay (Goodell *et al*., 2006). Further details of the chemical analyses are given in Rineau *et al*. (2012).

### Transcriptome analysis

After the incubation, the mycelium was collected, immediately frozen in liquid N_2_ and subsequently ground to a fine powder with a mortar. Total RNA was isolated using the RNeasy Plant Mini Kit (Qiagen, Hilden, Germany) with the RLC buffer and on‐column DNase treatment, according to the manufacturer's instructions. Total RNA was eluted in H_2_O and stored at −20°C until use. For quality assessments, all samples were inspected with an RNA 6000 Nano Kit on a 2100 Bioanalyzer (Agilent, Santa Clara, CA, USA). After reverse transcription into double‐stranded cDNA and barcoding by means of the massively parallel signature sequencing protocol (Brenner *et al*., 2000), libraries were sequenced (RNA‐Seq) using a HiSeq2000 instrument (Illumina Inc., San Diego, CA, USA) in single‐read mode and with a read length of 50 bp (IGA Technology Services, Udine, Italy; http://www.igatechnology.com). The reads were mapped onto the corresponding genomes by means of tophat (v.2.0.8b) (Kim *et al*., 2013). The transcript abundances were normalized using the R package DESeq (Anders & Huber, 2010) for each fungus by dividing the expression values by size factors to adjust for different sequence depth between the two conditions. The size factor for each sample was calculated as follows: the read count of each gene was divided by the geometric mean of the expression across the SOM and MMN samples. Significantly differentially expressed genes (SOM vs MMN) were identified with DESeq (*t*‐test, false discovery rate, *q*‐value < 0.01; Benjamini & Hochberg, 1995). The RNA‐Seq data are deposited in the GEO database (http://www.ncbi.nlm.nih.gov/geo/) with accession number GSE64897.

### Annotations

Gene models and annotations for each of the fungal species were retrieved from the Joint Genome Institute (JGI) MycoCosm database (Table S1). The signalp algorithm (v.4.0) was used for additional searches for prediction of secretion signals (Petersen *et al*., 2011). Conserved protein domain families (Pfam) were identified by searching through the Pfam family protein database (pfam_scan.pl tool with default settings) (Finn *et al*., 2014). Orphans were defined as sequences not having matches in the Pfam database or homologues in other organisms as revealed by BLASTP searches (Altschul *et al*., 1990) against the UniProt database (cut‐off 1E< 10^−5^). Putative peptidases were annotated by searching the proteome of each fungus against the MEROPS database (Rawlings *et al*., 2012).

Annotations on gene models encoding enzymes active on carbohydrates (CAZymes) were retrieved from the Carbohydrate‐Active Enzymes database (CAZy; http://www.cazy.org/), including the extended set of auxiliary activities (AAs) to cover redox enzymes that act in conjunction with CAZymes (Cantarel *et al*., 2009; Levasseur *et al*., 2013). Annotations for various types of PODs in all nine genomes were retrieved from the PeroxiBase database (Fawal *et al*., 2013). Gene models representing tyrosinases were retrieved directly from the JGI database by the use of InterPro term IPR002227 as a search term. All gene models of CAZymes, auxiliary activities, PODs and tyrosinases detected were inspected manually, and the selected and filtered gene model in the JGI database was modified if necessary. Genes encoding multidomain natural‐product‐biosynthesis enzymes (polyketide synthases, nonribosomal peptide synthetases and related enzymes) were annotated in a two‐step process: first, on the basis of homology by means of BLAST and a conserved domain search (Marchler‐Bauer *et al*., 2011) and, second, by manual inspection of each filtered gene model.

### Identification of orthologues and phylogenetic analyses

Orthologues were identified with the proteinortho program (Lechner *et al*., 2011). Principal component analysis (PCA) of orthologues was performed with the qlucore explorer software (v.2.2; Qlucore AB, Lund, Sweden) with default settings. For rescaling of the gene expression data before the PCA, clustering analyses, and phylogenetic analyses, the expression values of the orthologues were normalized by means of the DESeq package (Anders & Huber, 2010) and were then log_2_(counts + 1) transformed.

The construction of a species tree for the nine species included in this study was based on a phylogenetic analysis of 3148 one‐to‐one (1 : 1) orthologues. These orthologues were aligned with Mafft (v.7.147) (Katoh & Standley, 2013) with default settings, and the resulting alignments were trimmed with Gblocks (v.0.91b) (Talavera & Castresana, 2007) and concatenated into a supermatrix. The protein model was determined with prottest (v.3.4) (Abascal *et al*., 2005) and raxml‐hpc (Stamatakis, 2006) with default settings, and the Protgammalgf model was chosen to reconstruct the phylogenetic tree with 1000 bootstrap replicates.

A gene expression tree of the 1 : 1 orthologues was constructed by means of the neighbour‐joining (NJ) method. The expression values of three biological replicates from the same putative 1 : 1 orthologues used for the species tree were normalized, by means of the DESeq package, to make the values comparable between species. The sample distances were calculated as Euclidean distances between the mean of the samples or alternatively, as (1 − P) where P is the Spearman's correlation coefficient between the sample expression profiles. The APE package in R was used to construct the NJ expression tree and to perform the bootstrap analysis with 1000 replicates (Paradis *et al*., 2004).

Proteins encoding laccases (CAZy subfamily AA1_1) in all nine fungal genomes were retrieved from the CAZy database (http://www.cazy.org/). The 72 proteins were aligned with mafft (v.7.147) (Katoh & Standley, 2013) and subsequently trimmed with gblocks (Talavera & Castresana, 2007) using options to reduce the stringency (–b1 = 2 –b2 = 2 –b3 = 8 –b4 = 5 –b5 = h –b6 = y). prottest (v.2.4) (Abascal *et al*., 2005) was used to identify the most suitable evolutionary model. On the basis of the prottest results, the Whelan and Goldman (WAG) protein substitution model with a gamma shape parameter and a proportion of invariant sites (P‐Invar) was chosen for the phylogenetic reconstruction using raxml with 1000 bootstrap replicates (Stamatakis, 2006). itol was used to visualize the tree and map the differential gene expression to the tree (Letunic & Bork, 2011). A phylogenetic analysis of the aspartate protease family was performed using the alignment of the Pfam domain PF00026 (eukaryotic aspartyl protease). In total, 323 proteins containing this domain were identified in the nine analysed basidiomycete genomes. The domain sequences were extracted and retrieved using hmmalign in the hmmer3 package (v.3.1b1) (Eddy, 2011), and the sequences were subsequently aligned with mafft (v.7.147). The alignment was further trimmed with gblocks with the settings described for the proteins encoding laccases. A phylogenetic reconstruction using raxml and the JTT model with 1000 bootstrap replicates was performed and the results were viewed with itol. The tree can be viewed online at http://itol.embl.de/external.cgi?tree=1302351965015151413446270.

## Results

### Chemical conversion of the SOM extract

In the presence of glucose, all of the sampled ECM fungi grew and assimilated C and N from the SOM extract (Table S2; Figs S1a,b). The FTIR spectra of the SOM extract before and after a 7‐d incubation with fungi showed marked differences, indicating significant conversion of the organic compounds (Fig. 2a). A PCA based on the FTIR spectra allowed us to group the replicates of each fungus and clearly separated the initial SOM extract from the material modified by the fungi (Fig. 2b). The first principal component (PC1) explained 76.2% of the total variability and separated the initial material from the incubated material. The FTIR spectra of the SOM extract were separated along the PC1 axis into a group showing spectral features dominated by polysaccharide bands and N–H bending bands (right) and a group showing spectral features dominated by carbonyl bands (left), which are indicative of oxidized organic matter (Fig. S2). The separation of the ECM species was associated with the amounts of C and N assimilated by the fungi (Fig. S1a). The second principal component (PC2) accounted for 12.2% of the total variance and separated the fungi into two groups: one group consisting of the long‐distance‐exploration ECM fungi (*P. involutus* and *S. luteus*) and the closely related saprotrophic fungus *Hydnomerulius pinastri* and a second group containing the remaining ECM and saprotrophic species. PC2 was dominated by strong positive bands at 1510 cm^−1^, which are indicative of aromatic ring compounds (Fig. S2). The spectra of the SOM extracts incubated with *Serpula lacrymans* deviated significantly from those of the other species along PC2, mainly as a result of the peak at 1300 cm^−1^, which was attributed to esters. The two ECM boletes (*P. involutus* and *S. luteus*) were grouped close together by both PC1 and PC2.

To compare the decomposition capacity of *P. involutus* and *S. luteus* with that of the saprotrophic species in more detail, we analysed the SOM extracts using py‐GC/MS (Fig. 2c; Table S3). For all of the tested fungi, the levels of the major classes of compounds present in the SOM decreased during incubation. Analysis of the pyrolysates related to lignin residuals revealed changes in their chemical structure during fungal growth; specifically, the ratio of guaiacylacetone to *trans‐*propenylguaiacol, which indicates the oxidation state of guaiacyl‐lignin building blocks, was higher in the pyrolysates from the SOM extract exposed to the ECM fungi *P. involutus* and *S. luteus* than in pyrolysates from SOM exposed to the saprotrophic BR Boletales species (*H. pinastri* and *Coniophora puteana*) and *J. argillacea* (Fig. 2c, inset).

In the absence of glucose, the FTIR spectra of the SOM extract incubated with *P. involutus*,* S. luteus*,* H. pinastri* and *C. puteana* were almost identical to the spectra of the initial material (Fig. S3). Minute changes were detected in the FTIR spectra of the SOM extract incubated with *C. puteana*, observed as an increase in absorbance in the carbonyl region at 1710 cm^−1^ accompanied by a decrease in absorbance in the spectral region at 1350–1450 cm^−1^. Thus, the decomposition of the SOM was stimulated by glucose amendment in both the ECM and the saprotrophic species.

After growth, extracellular iron‐reducing activity was detected in the SOM extract of all fungi except *H. cylindrosporum* (Fig. S4). Thus, during growth on the SOM extract, iron‐reducing metabolites or iron‐reducing enzymes were produced by most of the examined ECM fungi.

### Global transcriptional responses

The transcriptional profiles of all fungi after growth on the SOM extract and on a mineral nutrient MMN medium were compared using RNA sequencing (Table S1). For comparison of the transcriptomes among the nine species, the genes were classified into orthologous groups (Table S4). In total, we identified 10 939 groups, of which 3148 were identified as putative 1 : 1 orthologues that were the best reciprocal BLAST hits shared by all nine species. The 1 : 1 orthologues constituted 14–25% of the total number of genes in each of the fungal genomes. The remaining genes were found in orthologous groups shared by two to eight species, in groups of so‐called co‐orthologues (i.e. genes that are duplicated within a lineage), or were species specific. Depending on the species, 16–29% of the transcripts were significantly up‐regulated in the SOM extracts (Table S1), and between 23 and 32% of those were 1 : 1 orthologues (Table S4).

To obtain an initial view of the expression patterns, we performed a PCA on the expression levels of the 1 : 1 orthologues. The PCA closely grouped the SOM‐ and MMN‐grown samples for each species (Fig. S5). PC1 (which explained 15% of the variation) clustered the Boletales species together and separated them from the species in the Jaapiales, Agaricales and Atheliales clades. PC2 (13% of the variation) separated the ECM fungi from the saprotrophs. To reconstruct the evolutionary trends in more detail, an NJ tree was constructed based on the distances of the expression levels of the 1 : 1 orthologues (Figs 1, S6). Consistent with the PCA results, the divergence of gene expression between a fungus grown on SOM extract and the same fungus grown on MMN medium was significantly less than the divergence between species, and the species were correctly separated into the four major clades. However, the topologies of the trees constructed from the expression data and from the sequence data (species tree) were not always consistent (Fig. 1). In agreement with more extensive genome phylogenies (Kohler *et al*., 2015), our species tree placed the ECM fungus *P. involutus* close to the BR species *H. pinastri*, whereas the expression tree clustered *P. involutus* and *S. luteus*.

**Figure 3:**
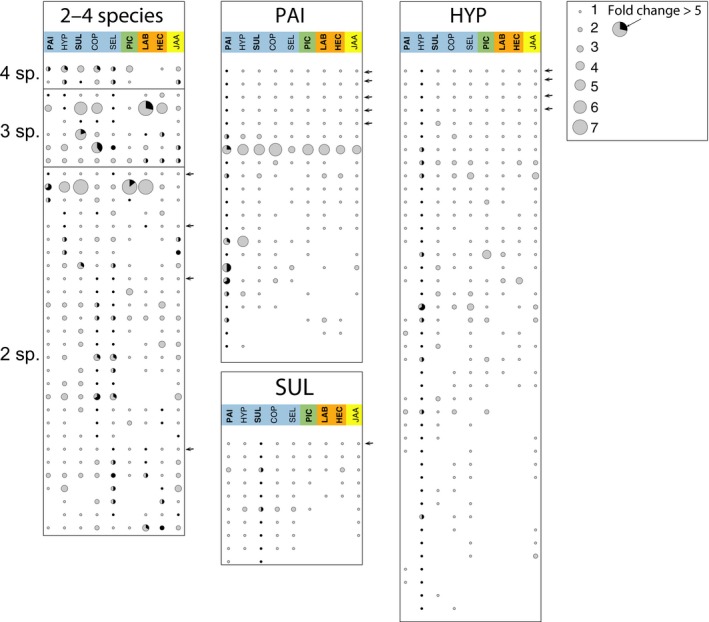
Phylogenetic distribution of soil organic matter (SOM)‐up‐regulated genes. The panel shows the expression and presence of SOM‐up‐regulated genes in orthologous groups (rows, fold change > 5 of pairwise comparisons in SOM extract vs modified Melin–Norkrans (MMN) medium; *q *< 0.01; *n* = 3) shared by at least two species (columns). The species abbreviations and clade affiliations (colour coded) are shown in the legend of Fig. 1. The size of the circles indicates the number of genes in the orthologous groups within a given species (if any) and the black slice is proportional to the number of up‐regulated genes. The arrows indicate 14 1 : 1 orthologues. The left panel shows the orthologous groups that were up‐regulated in two to four species (sp.), and the middle and right panels show orthologous groups that were up‐regulated uniquely in *Paxillus involutus* (PAI), *Hydnomerulius pinastri* (HYP) and *Suillus luteus* (SUL). Orthologous groups that were uniquely up‐regulated in the other species are shown in Supporting Information Fig. S7. Annotations of orthologue clusters are shown in Table S6.

Analysis of the most highly up‐regulated genes during SOM decomposition showed that a majority of them were found in orthologue clusters whose members were only up‐regulated in a single species, or they were nonorthologues genes. We identified 715 genes that were at least five‐fold SOM‐up‐regulated (pairwise comparisons in SOM extract vs MMN medium; *q *< 0.01; *n* = 3) in the nine analysed species. Of those genes, 387 were identified as having orthologues in at least two species, and 328 were found to be species specific (Table S5). The up‐regulated orthologues were found in 321 orthologue clusters. None of these clusters contained genes that were up‐regulated in all species. Genes from two clusters were up‐regulated in four species, those from six clusters in three species and those from 28 clusters in two species, and the remaining 285 orthologue clusters contained up‐regulated genes from one species only (Figs 3, S7). The highly SOM‐up‐regulated orthologue clusters encoded a diverse set of protein families (Table S6). Out of the 241 unique Pfam families that were identified in these orthologues, none were up‐regulated in all species. In total, 22 Pfam families were commonly up‐regulated in at least three species including domains of transporters, proteases, oxidoreductases and glycoside hydrolases (Table S7).

### Secretome

**Figure 4:**
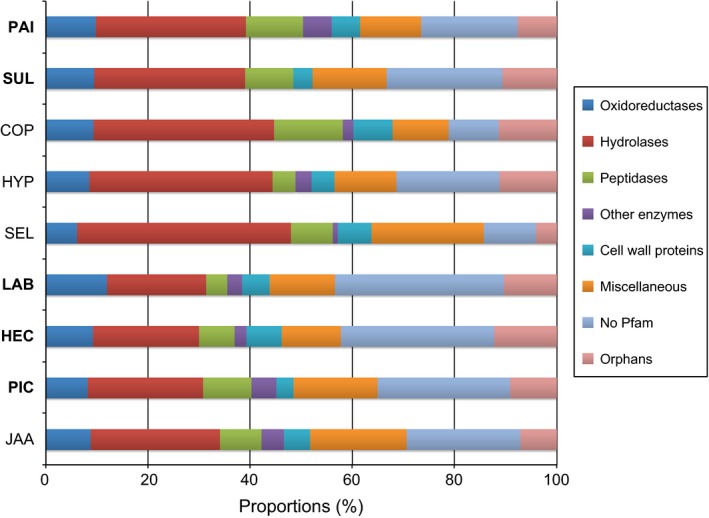
Functional categories of genes encoding secreted proteins and which are up‐regulated during growth on soil organic matter (SOM) extract. Shown is the proportion of proteins annotated as oxidases, hydrolases, peptidases, other enzymes, and cell wall proteins that were significantly up‐regulated as revealed by RNA‐Seq analysis (pairwise comparisons in SOM extract vs modified Melin–Norkrans (MMN) medium; *q *< 0.01; *n* = 3). The category ‘Miscellaneous’ includes proteins with a diverse set of conserved protein family (Pfam) domains that could not be annotated as enzymes or cell wall proteins. Orphans are putative proteins that lack both Pfam domains and homologues. The species abbreviations are listed in the legend of Fig. 1. The total numbers of SOM‐up‐regulated genes are shown in Supporting Information Table S1.

Between 6 and 9% of all SOM‐up‐regulated genes (*q *< 0.01) were predicted to encode secreted proteins (Table S1). The up‐regulated secretome contained a large fraction of genes that were either species specific or shared with a limited set of other species (Tables S4). Still, the functional annotation terms of the proteins in the up‐regulated secretome were similar. The SOM‐induced secretomes contained a large fraction (38–60%) of genes encoding extracellular enzymes such as oxidases, hydrolases and peptidases (Fig. 4). Apart from these enzymes, the up‐regulated secretomes also contained cell wall proteins including members of the hydrophobin family (Pfam domain PF01185). Hydrophobins are small (*c*. 100 amino acid (aa) residues) cysteine‐rich proteins that can self‐assemble and form aggregations of proteins on surfaces of filamentous ascomycetes and basidiomycetes (Wösten, 2001). Among the most highly expressed proteins in the up‐regulated secretome were also a large number of small secreted proteins (SSPs; < 300 aa) that lacked both Pfam domains and homologues, so they can be referred to as orphans.

### Lignin‐ and carbohydrate‐degrading enzymes

**Figure 5:**
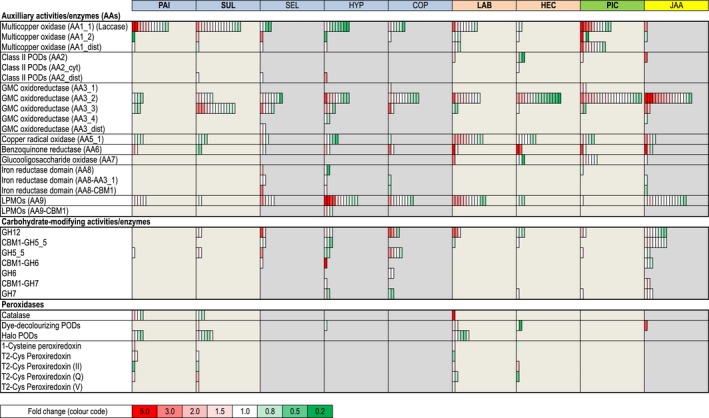
Soil organic matter (SOM) regulation of selected genes encoding auxiliary redox activities/enzymes (AAs), peroxidases and carbohydrate‐modifying enzymes acting on cellulose. Shown is the average ratio of gene expression (*n* = 3) of pairwise comparisons in SOM extract vs modified Melin–Norkrans (MMN) medium. Within each subpanel, one for each species, the boxes represent individual gene models found within the family, and the colours show the fold change in expression. The complete sets of enzymes are shown in Supporting Information Figs S8–S10 and Tables S8–S10. The species abbreviations and clade affiliations (colour coded) are shown in the legend of Fig. 1.

Upon SOM decomposition, all of the examined ECM fungi expressed a range of genes encoding enzymes involved in the oxidative degradation of lignocellulose and the production of hydrogen peroxide: for example, laccases, which catalyse the oxidation of a wide range of organic compounds; glucose‐methanol‐choline oxidoreductases (GMCOs) and copper‐radical oxidases (CROs), which reduce oxygen to peroxide and might support Fenton chemistry and PODs by supplying extracellular hydrogen peroxide; various PODs; and tyrosinases (Fig. 5; Tables S8, S9).

Distinctive differences in the expression patterns of oxidoreductases were found between the ECM fungi and the saprotrophic Boletales (Figs 5, S8). Compared with the BR species, *P. involutus* and *S. luteus* expressed more laccases and several of them were significantly up‐regulated in the SOM extract. Moreover, *P. involutus* and *S. luteus* expressed several genes encoding PODs such as catalases, heme‐containing haloperoxidases and one class of nonheme typical two‐cysteine peroxiredoxin that was not found in the saprotrophic Boletales (Fig. S9; Table S9). By contrast, the BR Boletales highly expressed various iron reductases (such as AA8) (Fig. 5).

*Laccaria bicolor*,* H. cylindrosporum* and *P. croceum* expressed unique profiles of oxidoreductases that differed from the profiles of *P. involutus* and *S. luteus* (Figs 5, S8, S9). For example, the SOM‐induced transcriptome of *L. bicolor* was characterized by CROs; that of *H. cylindrosporum* by low numbers and expression levels of laccases (AA1_1); and that of *P. croceum* by a comparatively large number of GMCOs and secreted tyrosinases. Although putative class II PODs of *L. bicolor*,* H. cylindrosporum* and *P. croceum* were up‐regulated on the SOM substrate, none of them was classified as classical class II PODs (a group that includes lignin PODs, manganese PODs and versatile PODs), which are typical for WR fungi (Table S8). The dye‐decolourizing PODs were also up‐regulated in *L. bicolor*,* H. cylindrosporum* and *S. luteus* (Fig. 5).

**Figure 6:**
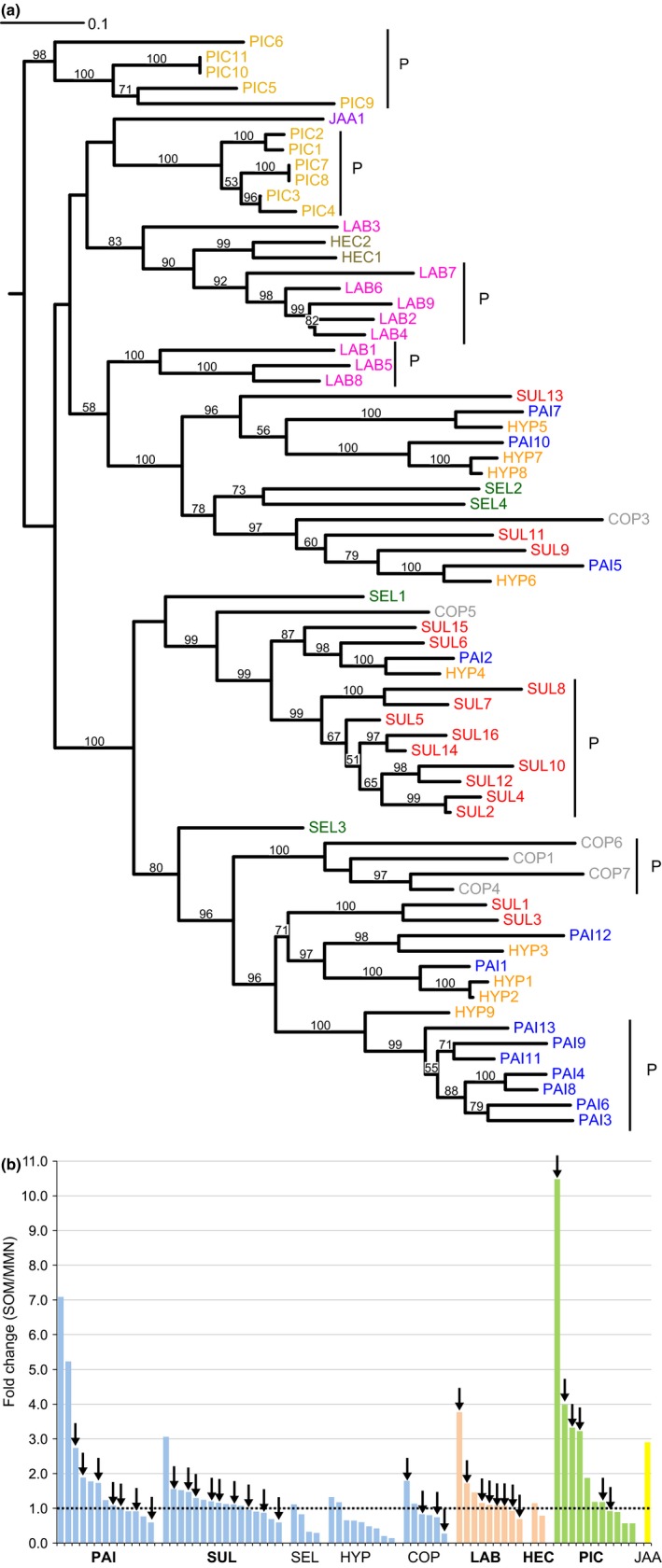
Phylogeny and expression patterns of laccases. (a) An unrooted maximum likelihood tree of protein sequences of 71 laccases retrieved from sequences of genome models (cf. Supporting Information Table S8). Bootstrap values are shown for branches having > 50% support. The gene models were assigned trivial names according to the fold change (from highest to lowest) in pairwise comparisons in soil organic matter (SOM) extract vs modified Melin–Norkrans (MMN) medium. Vertical bars labelled with a ‘P’ indicate paralogue clades with > 50% bootstrap support grouping at least three sequences coming from only one species. (b) SOM regulation of the 71 laccase genes. The bars show the average fold change (*n *= 3) in pairwise comparisons in media containing SOM extract vs MMN medium. Along the *x*‐axis are gene models from the different fungi. For each species, the models are arranged from highest to lowest fold changes. The arrows indicate that the gene is found in a clade of paralogues. A fold change value of 1 indicates equal transcription level in the two media. The species abbreviations and clade affiliations (colour coded) are shown in the legend of Fig. 1.

Many of the enzymes that were up‐regulated upon SOM decomposition are members of large gene families including laccases (Fig. 5). Phylogenetic analysis showed that the laccase family in *P. involutus*,* S. luteus*,* L. bicolor* and *P. croceum* contained many recent gene duplicates (i.e. paralogues) (Fig. 6a). Many of them were up‐regulated during growth on the SOM extract (Fig. 6b).

The saprotrophic species, as was expected, expressed several genes encoding GHs and carbohydrate esterases (CEs) that are predicted to be active on cellulose, cellulose/hemicelluloses, hemicelluloses and starch (Figs 5, S10a; Table S10). By contrast, the ECM expressed a limited set of genes encoding CAZymes and only a few of them were SOM‐up‐regulated (Fig. S10b). CAZy genes induced by the addition of SOM extract differed between ECM species. Among the induced genes were LPMOs (formerly GH61), which catalyse the oxidative cleavage of cellulose (*L. bicolor*), GH12 endoglucanases (*P. croceum* and *L. bicolor*), a GH5‐7 mannanase (*S. luteus*), GH79 β‐glucuronidases, GH3 β‐glucosidases, which are involved in the cleavage of cellobiose to glucose, and various other hemicellulose‐ and starch‐related genes.

### Peptidases

Depending on the species, 4–13% of the SOM‐induced secretome constituted peptidases (Fig. S11; Table S11). Although the most common types were endopeptidases of the aspartate protease A1 subfamily, each species expressed a diverse set of endopeptidases (including subfamilies M36, S8 and S53) and exopeptidases (M28, S9 and S28). The variation in the expression patterns of peptidases among species was smaller than that of oxidoreductases. A phylogenetic analysis of the A1 aspartate protease family showed that this family contained both gene duplicates shared with other species (i.e. orthologues) as well paralogues. Members of both categories were SOM‐up‐regulated (Fig. S12; Table S12).

### Secondary metabolites

All nine examined fungi contained genes encoding enzymes with possible roles in the synthesis of secondary metabolites (Table S13). The total number of polyketide synthase and nonribosomal peptide synthetase genes varied from three to four in *L. bicolor* and *P. croceum* to 16 in *S. lacrymans*, and several of those genes were up‐regulated upon SOM decomposition (Fig. S13). With the exception of *C. puteana*, the Boletales species contained genes encoding quinone synthetases. Two of these genes were significantly up‐regulated in *P. involutus*, three were up‐regulated in *S. luteus* and one was up‐regulated in *H. pinastri*.

## Discussion

In one of the earliest articles on ECM symbiosis, Frank stated that there is ‘no doubt that mycorrhizal fungi account for a major fraction of litter turnover, in order to supply nutrients to the trees’ (translation from German) (Frank, 1894). However, ECM fungi have primarily been considered as biotrophs that obtain their energy and C from the host plant and have a limited capacity to affect the decomposition of organic material, and the decomposition of such material has been assumed to be carried out almost exclusively by free‐living saprotrophic fungi. The limited decomposing ability of ECM fungi is supported by recent findings showing that they have lost many of the genes that encode plant cell wall‐degrading enzymes in their saprotrophic ancestors (Kohler *et al*., 2015). However, here we demonstrate that ECM fungi representing at least four distant origins of symbiosis have retained a significant capacity to decompose SOM using oxidative mechanisms. Although recent laboratory and field studies suggest that ECM fungi can act as SOM decomposers (Rineau *et al*., 2012; Bödeker *et al*., 2014; Phillips *et al*., 2014; Lindahl & Tunlid, 2015), we show that this capacity is widespread in ECM fungi, expanding the roles of these fungi in the forest C cycle.

Spectroscopic analysis and genome‐wide transcriptome profiling showed that the decomposition of SOM by the analysed ECM fungi involved oxidative processes and that these processes were similar to those of saprotrophic fungi. The chemical changes in the organic material were reflected in marked modifications of the polysaccharide and carbonyl regions of the FTIR spectra. In particular, the decrease in the polysaccharide content coincided with an increase in C=O linkages, indicating partial oxidation of the material during its decomposition (Weiland & Guyonnet, 2003). The SOM decomposed by species within the Boletales clade was analysed using py‐GC/MS, which showed that the ECM species modified the side‐chain structures of lignin‐derived molecules, as expected by a Fenton mechanism similar to that of BR saprotrophs (Yelle *et al*., 2011). The detection of extracellular iron‐reducing activity further supported a Fenton mechanism (Hatakka & Hammel, 2010; Eastwood *et al*., 2011). Notably, all ECM species except *H. cylindrosporum* had extracellular iron‐reducing activity upon growth on the SOM extract, which agrees with experimental studies showing that Fenton chemistry is present in Agaricomycetes outside BR fungi (Tanaka *et al*., 2007; Gomez‐Toribio *et al*., 2009; Arantes *et al*., 2011). During SOM decomposition, the ECM fungi expressed a number of oxidoreductases (e.g. GMCOs and CROs) that are thought to be involved in generating H_2_O_2_ in Fenton‐type reactions in BR fungi (Martinez *et al*., 2009; Levasseur *et al*., 2013). Genes encoding enzymes more frequently connected with lignin decay in WR fungi were also expressed, including laccases, dye‐decolourizing PODs and haloperoxidases (Hatakka & Hammel, 2010; Hofrichter *et al*., 2010).

Though the observed oxidative modifications of the SOM during decomposition were similar across the examined ECM species, the extent of the oxidation differed and was related to the growth rate and the rate of N uptake by the ECM fungi. The rapidly growing, long‐distance‐exploration types oxidized SOM and assimilated N to a greater extent than did the slow‐growing, short‐ and medium‐distance‐exploration types. Differences in N uptake between exploration types have been observed in field studies using isotopes (Hobbie & Agerer, 2010). Despite the similarity in the chemical transformation of the SOM extract, each species expressed a different set of transcripts, which suggests that the decomposition mechanisms have diverged over evolutionary time. For the most part, the divergence in the SOM‐induced expression profiles increased with evolution: that is, closely related species displayed profiles that were more similar than distantly related species. This is expected not only because of the evolutionary distance between the examined ECM lineages but also because of the diverse nutritional backgrounds from which these ECM lineages have evolved (Matheny *et al*., 2006; Kohler *et al*., 2015). Analysis of the phylogenetic distribution of the SOM‐up‐regulated genes indicates that the diversification of the decomposition mechanism involves several evolutionary processes. First, diversification is associated with differences in the regulation of orthologues genes that are shared between distantly related species. Second, the decomposition mechanism has evolved by incorporating enzymes from different families with apparently similar catalytic properties. For example, the examined species expressed genes encoding various enzymes from the laccase, GMCO and CRO gene families that all could presumably generate the H_2_O_2_ that is needed for Fenton chemistry. Third, diversification is associated with the expression of species‐specific genes. Such genes include recent gene duplicates (i.e. paralogues) of gene families that are conserved across the Agaricomycetes, as exemplified by the laccase gene family, but also orphans which lack both Pfam domains and homologues in other species.

The observed species‐specific changes in gene expression levels within the Boletales suggest that selection may shape oxidative decomposition mechanisms over a short evolutionary time. Whether *P. involutus* and *S. luteus* represent two independently evolved ECM lineages, or a single ECM origin and a reversal to saprotrophy in *H. pinastri*, is not clear (Kohler *et al*., 2015). Hence, the similar oxidative decomposition mechanisms of *P. involutus* and *S. luteus* might be a result of convergent evolution from two independent saprotrophic ancestors. Alternatively, if the ECM lifestyle evolved only once between *P. involutus* and *S. luteus*, then the saprotrophic decomposition mechanism of *H. pinastri* represents a reversal from an ECM ancestor. Such an ancestor probably contained a larger array of genes encoding saprotrophy‐related enzymes such as LPMOs or GH28 pectinases than do the extant ECM Boletales (Kohler *et al*., 2015).

The extensive SOM‐induced expression of laccases and the expansion of this family in the genomes of ECM fungi as compared with that in the BR wood decayers suggest that laccases play an important role in the decomposition of SOM. This possibility is supported by the fact that laccases have low redox potentials that are suitable for oxidizing small lignin‐ and phenolic‐like compounds present in SOM (Martinez *et al*., 2005; Hatakka & Hammel, 2010). By contrast, in the absence of low‐molecular‐weight redox mediators, laccases cannot depolymerize macromolecular lignin found in native wood substrates (Martinez *et al*., 2005; Hatakka & Hammel, 2010). However, in addition to their possible involvement in the degradation of organic material, laccases may also have several other functions in fungi, for example in pigment formation, fruiting body development, defence reactions and detoxification (Kües & Ruhl, 2011).

An implication from this study is that the presently used methods for measuring the decomposition activities of ECM fungi in the field that are based on measuring enzyme activities or transcript levels of oxidases such as laccases, phenol oxidases and class II PODs (e.g. Bödeker *et al*., 2014; Phillips *et al*., 2014) do not capture the diversity of oxidative mechanisms. Our results suggest that ECM fungi decompose SOM using mechanisms involving the action of both oxidative enzymes and oxygen radicals formed by nonenzymatic Fenton reactions. Further studies are needed to identify the key enzymes and molecular components of these oxidation reactions, and how their expression levels correlate with the chemical modifications of SOM. Data from such experiments will probably generate novel biomarkers that can accurately predict the decomposition activities of ECM fungi *in situ*.

In agreement with previous studies, addition of glucose stimulated the oxidative decomposition of organic matter in both ECM fungi (Rineau *et al*., 2012) and BR fungi (Varela *et al*., 2003). Accordingly, radical‐based oxidation mechanisms in ECM and saprotrophic fungi can be described as co‐metabolic processes that require growth on a source of utilizable C and energy. In saprotrophs, metabolic C is liberated from dead organic matter by the action of hydrolytic enzymes, such as CAZymes. Polysaccharides were decomposed by *H. pinastri* and *C. puteana* during growth on the SOM extract (Fig. 2c). However, the very low extent of oxidation of the SOM extract in the absence of glucose suggests that the amount of C that was released during the limited time of incubation (7 d) was too low to support significant levels of oxidation. In ECM fungi, metabolic C is primarily provided by the plant host and the overall reduced number of genes coding for cell‐wall‐degrading enzymes in ECM species (Kohler *et al*., 2015) seems to support this. This hypothesis is also supported by field studies showing that ECM root tips only accumulate trace levels of ^14^C‐labelled leaf litter (Treseder *et al*., 2006). That ECM fungi obtain most of the C from their host plant is also indicated by ^14^C dating showing that the structural C was synthesized from recent photosynthate while proteins of ECM sporocarps were built from older pools of soil organic N (Hobbie *et al*., 2013). In addition, progressive increases in C : N ratios during ECM colonization of organic matter support selective assimilation of N (Lindahl *et al*., 2007; Clemmensen *et al*., 2013). However, upon growth on the SOM extract, the ECM species in our data set expressed low levels of a few CAZymes (e.g. mannanases and endoglucanses) involved in plant cell wall decomposition. This suggests that some hydrolytic decomposition of carbohydrates could have taken place during SOM decomposition. In saprophytic fungi, the expression of plant cell wall‐degrading CAZymes is commonly repressed in the presence of glucose (Aro *et al*., 2005). Additional studies are needed to show if such enzymes are repressed in a similar way in ECM fungi and if their activity can release metabolic C during SOM decomposition.

Taken together, the results of this study suggest that the primary function of the oxidative decomposition system of ECM fungi is to open up SOM complexes and thereby mobilize sequestered proteins and other nutrient sources. However, the oxidation may also influence the functionality of the SOM and the stability of soil C. Oxidative transformations can make the SOM available for further degradation by saprotrophic microorganisms that contain the enzymatic machinery for depolymerization and complete mineralization. Alternatively, oxidation of biomolecules present in the aqueous pool of SOM will enhance their polarity and chemical reactivity, and such modifications could promote the formation of stable supramolecular aggregates or organic matter–mineral complexes (Kleber & Johnson, 2010; Kleber *et al*., 2015). These processes and the widespread presence of ECM fungi (Högberg & Högberg, 2002) suggest that ECM fungi can have an important role in the turnover and stabilization of organic matter in forest soils.

## Author contributions

F.S., C.N., T.J., P.P. and A.T. designed the research; F.S., C.N., M.E., F.R., M.S., R.C., T.J., P.P. and A.T. performed the research; F.S., C.N., J. Braesel, M.S., F.R., B.C., D.F., G.L., J. Bentzer, D.H., B.H., D.A., T.J., D.S.H., F.M., P.P. and A.T. analysed the data; F.S., C.N., D.F., T.J., P.P. and A.T. wrote the paper.

## Supporting information

Please note: Wiley Blackwell are not responsible for the content or functionality of any supporting information supplied by the authors. Any queries (other than missing material) should be directed to the *New Phytologist* Central Office.

**Fig. S1** Assimilation of C and N by the fungi during growth on SOM extract.
**Fig. S2** PCA loadings of the FTIR spectra of the SOM.
**Fig. S3** The effects of glucose on SOM decomposition by *Coniophora puteana*,* Hydnomerulius pinastri*,* Paxillus involutus* and *Suillus luteus*.
**Fig. S4** Iron‐reducing activity produced during growth on SOM extracts.
**Fig. S5** PCA based on the expression patterns of orthologues.
**Fig. S6** Neighbour‐joining gene expression trees.
**Fig. S7** Phylogenetic distribution of SOM‐up‐regulated genes.
**Fig. S8** Expression profiles of genes encoding AAs.
**Fig. S9** Expression profiles of genes encoding peroxidases and tyrosinases.
**Fig. S10** Expression profiles of genes encoding selected CAZymes.
**Fig. S11** Expression profiles of genes encoding extracellular peptidases.
**Fig. S12** Phylogeny and expression patterns of aspartate proteases.
**Fig. S13** Expression profiles of selected genes encoding biosynthetic enzymes involved in secondary metabolism.
**Table S1** Fungi used in this study and the assessment of the transcribed fraction of their genomes based on RNA‐Seq data
**Table S2** Carbon and nitrogen concentrations of the SOM extract
**Table S3** List of pyrolytic compounds identified by py‐GC/MS analysis of the SOM extract
**Table S4** Numbers of gene models in various (co)‐orthologous groups
**Table S5** Numbers of highly SOM‐up‐regulated genes
**Table S6** Annotation of highly SOM‐up‐regulated orthologues
**Table S7** Protein families found among the highly SOM‐up‐regulated orthologues

**Table S8** Gene models of AAs
**Table S9** Gene models of peroxidases and tyrosinases
**Table S10** Gene models of CAZymes
**Table S11** Gene models of peptidases
**Table S12** Gene models encoding aspartate peptidases
**Table S13** Gene models of natural‐product biosynthesis enzymes
