# Supplementary material for: Ectomycorrhizal fungi decompose soil organic matter using oxidative mechanisms adapted from saprotrophic ancestors
Source: New Phytol. 2015 Nov 3;209(4):1705–19. doi: 10.1111/nph.13722 (PMC5061094; doi:10.1111/nph.13722)
Supplement: Supplementary file 1 — Fig. S1 Assimilation of C and N by the fungi during growth on SOM extract. Fig. S2 PCA loadings of the FTIR spectra of the SOM. Fig. S3 The effects of glucose on SOM decomposition by Coniophora puteana, Hydnomerulius pinastri, Paxillus involutus and Suillus luteus. Fig. S4 Iron‐reducing activity produced during growth on SOM extracts. Fig. S5 PCA based on the expression patterns of orthologues. Fig. S6 Neighbour‐joining gene expression trees. Fig. S7 Phylogenetic distribution of SOM‐up‐regulated genes. Fig. S8 Expression profiles of genes encoding AAs. Fig. S9 Expression profiles of genes encoding peroxidases and tyrosinases. Fig. S10 Expression profiles of genes encoding selected CAZymes. Fig. S11 Expression profiles of genes encoding extracellular peptidases. Fig. S12 Phylogeny and expression patterns of aspartate proteases. Fig. S13 Expression profiles of selected genes encoding biosynthetic enzymes involved in secondary metabolism. Table S1 Fungi used in this study and the assessment of the transcribed fraction of their genomes based on RNA‐Seq data Table S2 Carbon and nitrogen concentrations of the SOM extract Table S3 List of pyrolytic compounds identified by py‐GC/MS analysis of the SOM extract Table S4 Numbers of gene models in various (co)‐orthologous groups Table S5 Numbers of highly SOM‐up‐regulated genes Table S6 Annotation of highly SOM‐up‐regulated orthologues Table S7 Protein families found among the highly SOM‐up‐regulated orthologues [file NPH-209-1705-s001.pdf]

***New Phytologist* Supporting Information**

Article title: **Ectomycorrhizal fungi decompose soil organic matter using oxidative mechanisms adapted from saprotrophic ancestors**

Authors: Firoz Shah, César Nicolás, Johan Bentzer, Magnus Ellström, Mark Smits, Francois Rineau, Björn Canbäck, Dimitrios Floudas, Robert Carleer, Gerald Lackner, Jana Braesel, Dirk Hoffmeister, Bernard Henrissat, Dag Ahrén, Tomas Johansson, David S. Hibbett, Francis Martin, Per Persson and Anders Tunlid

Article acceptance date: 22 September 2015

The following Supporting Information is available for this article:

Fig. S1 Assimilation of C and N by the fungi during growth on SOM extract.

Fig. S2 PCA loadings of the FTIR spectra of the SOM.

Fig. S3 The effects of glucose on the SOM decomposition by *C. puteana*, *H. pinastri*, *P. involutus* and *S. luteus*.

Fig. S4 Iron-reducing activity produced during growth on SOM extracts.

Fig. S5 PCA based on the expression patterns of orthologues.

Fig. S6 Neighbour-joining gene expression trees.

Fig. S7 Phylogenetic distribution of SOM-upregulated genes.

Fig. S8 Expression profiles of genes encoding auxiliary redox activities/enzymes (AAs).

Fig. S9 Expression profiles of genes encoding peroxidases and tyrosinases.

Fig. S10 Expression profiles of genes encoding selected carbohydrate-modifying enzymes (CAZymes).

Fig. S11 Expression profiles of genes encoding extracellular peptidases.

Fig. S12 Phylogeny and expression patterns of aspartate proteases.

Fig. S13 Expression profiles of selected genes encoding biosynthetic enzymes involved in secondary metabolism.

Table S1 Fungi used in this study and the assessment of the transcribed fraction of their genomes based on RNA-Seq data.

Table S2 Carbon and nitrogen concentrations of the SOM extract.

Table S3 List of pyrolytic compounds identified by py-GC/MS analysis of the SOM extract.

Table S4 Numbers of gene models in various (co)-orthologous groups.

Table S5 Numbers of highly SOM-upregulated genes.

Table S6 Annotation of highly SOM-upregulated orthologues.

Table S7 Protein families found among the highly SOM-upregulated orthologues.

Table S8 Gene models of auxiliary redox enzymes (AAs). (Excel file)

Table S9 Gene models of peroxidases and tyrosinases. (Excel file)

Table S10 Gene models of CAZymes. (Excel file)

Table S11 Gene models of peptidases. (Excel file)

Table S12 Gene models encoding aspartate peptidases. (Excel file)

Table S13 Gene models of natural-product biosynthesis enzymes. (Excel file)

Fig. S1 Assimilation of C and N by the fungi during growth on SOM extract. (**A**) Correlation of C and N uptake based on the TOC/TN content (*c.f.* Table S2). Bars indicate ± SE (*n*=3). The species abbreviations are listed in Table S1. (**B**) The increase in radial growth of the mycelia during the experiment. *L. bicolor* and *J. argillacea* were growing into the medium, whereas the mycelia of all other fungi were growing on the surface of the glass beads. *S. lacrymans* grew as a thin and rapidly expanding mycelium.

(A)

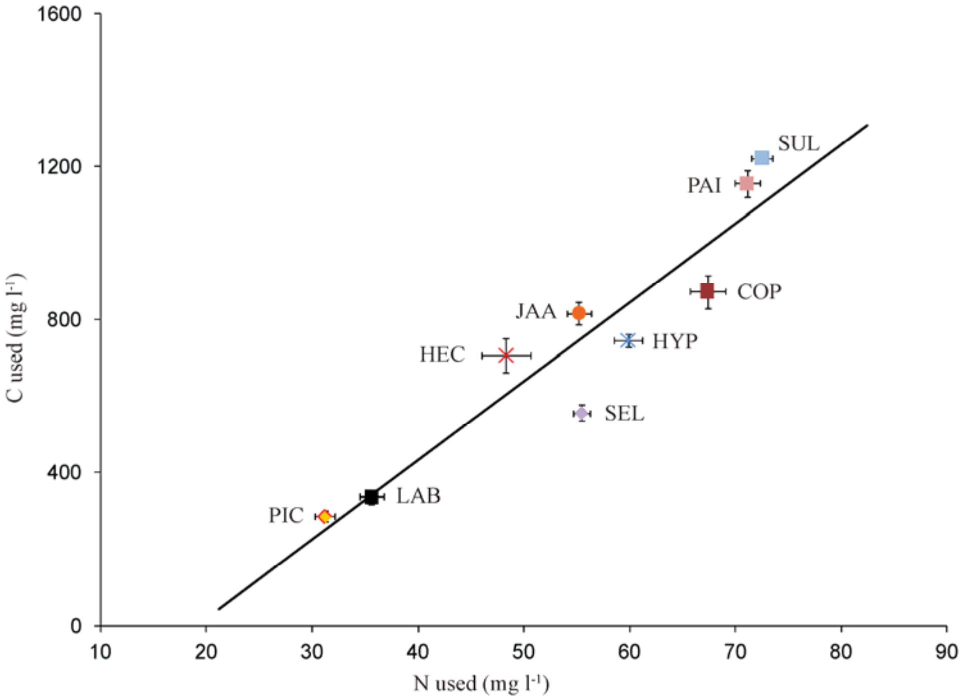

(B)

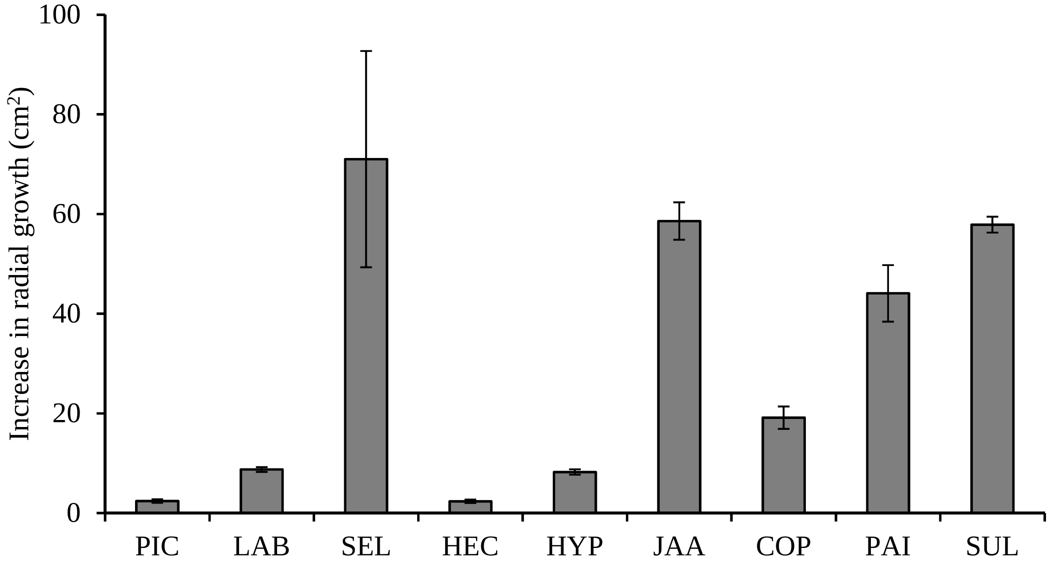

Fig. S2 PCA loadings of the FTIR spectra of the SOM extract. Shown are the PCA loadings of the FTIR spectra of the SOM extract before the inoculation (initial material) and after 7 days of incubation with different ECM and saprophytic fungi. PC1 and PC2 refer to principal components 1 and 2, respectively. Refer to main text and Fig. 2a and 2b for more details.

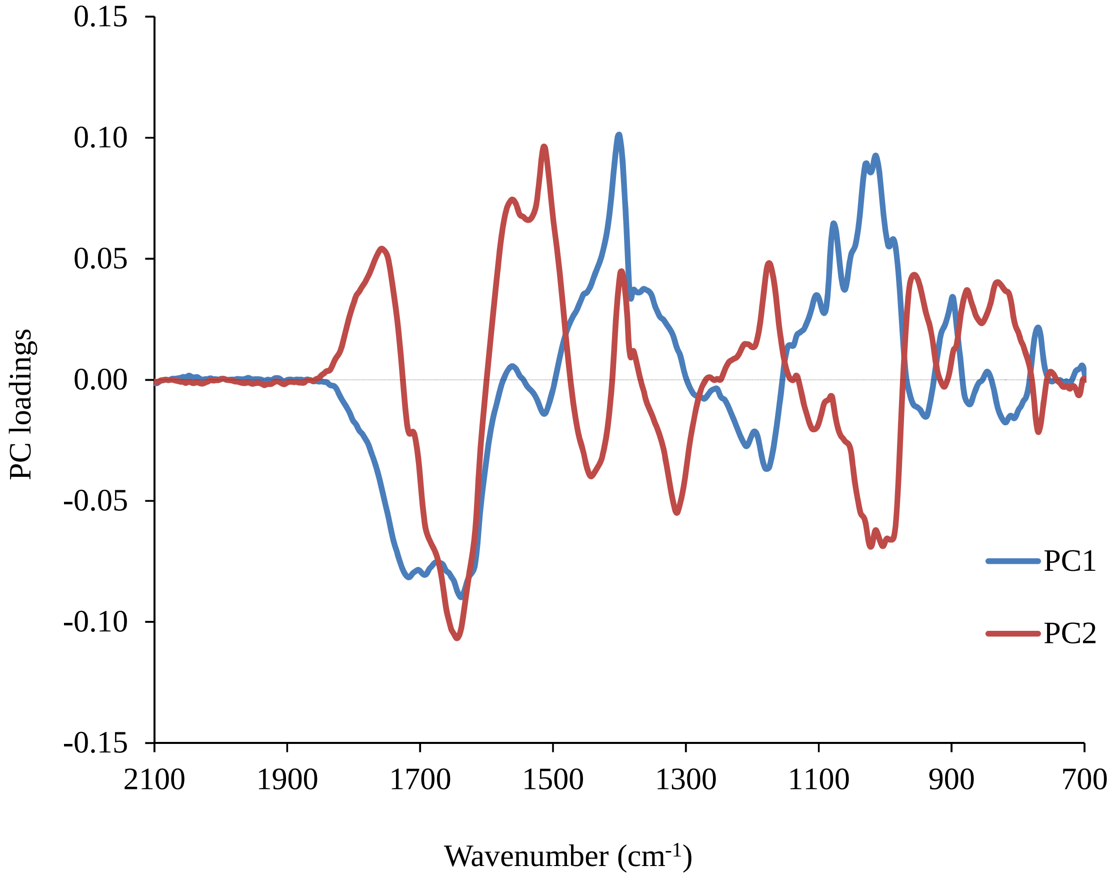

Fig. S3 The effects of glucose on the SOM decomposition by *C. puteana*, *H. pinastri*, *P. involutus* and *S. luteus*. The figure shows the FTIR spectra of the SOM extract without glucose being supplemented, before (green line) and after 7 days of incubation (red line). All spectra have been normalized to the same total area over the wavenumber region displayed (*n*=3). Spectral changes in the SOM extracts were not in general marked after the incubation with fungi, although those incubated with *C. puteana* showed an increase of the carbonyl region at 1710 cm^{-1} accompanied by a decrease of the spectral region at 1350 - 1450 cm^{-1}.

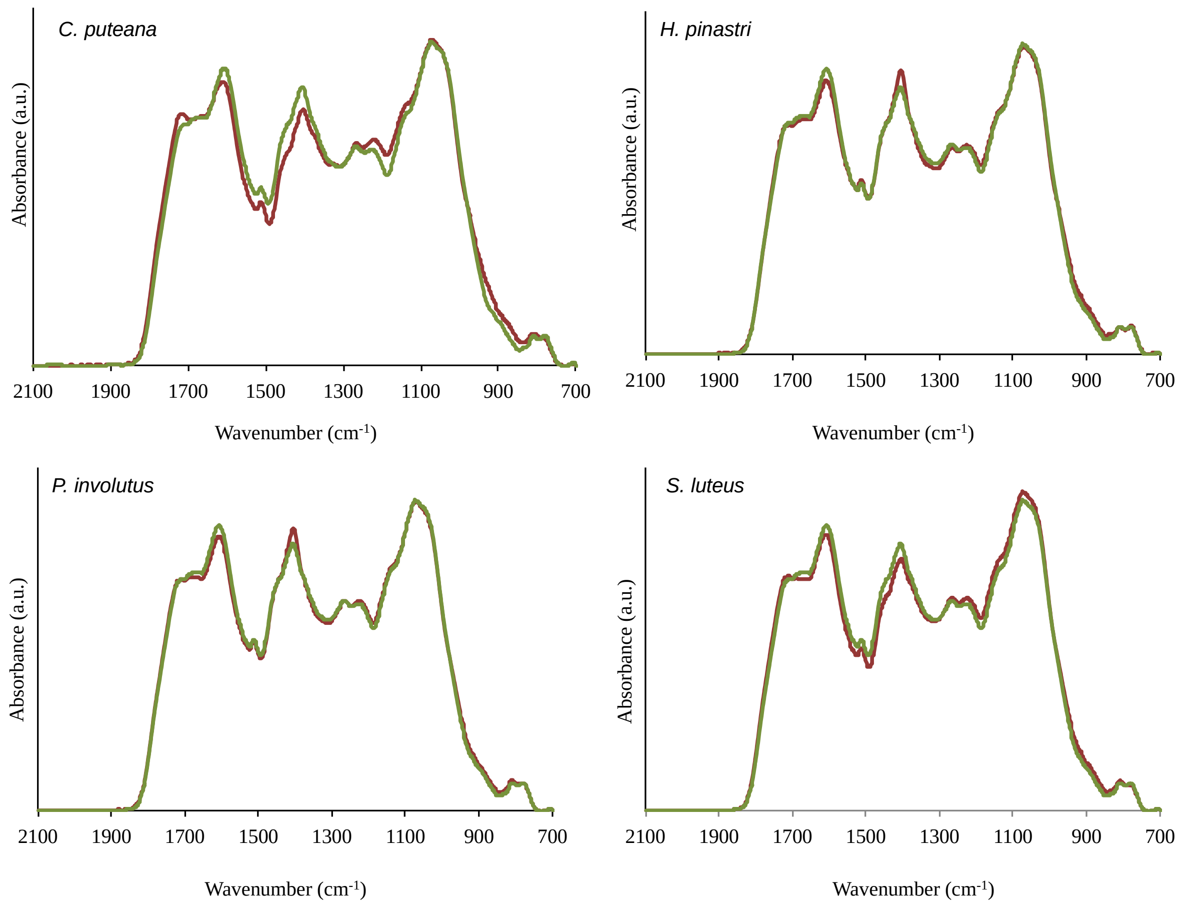

Fig. S4 Iron-reducing activity produced during growth on SOM extracts. Ferrozine assay measurement of SOM extract after incubation with 9 fungal species for 7 days. Bars indicate ± SE (*n*=3). The species abbreviations are listed in Table S1.

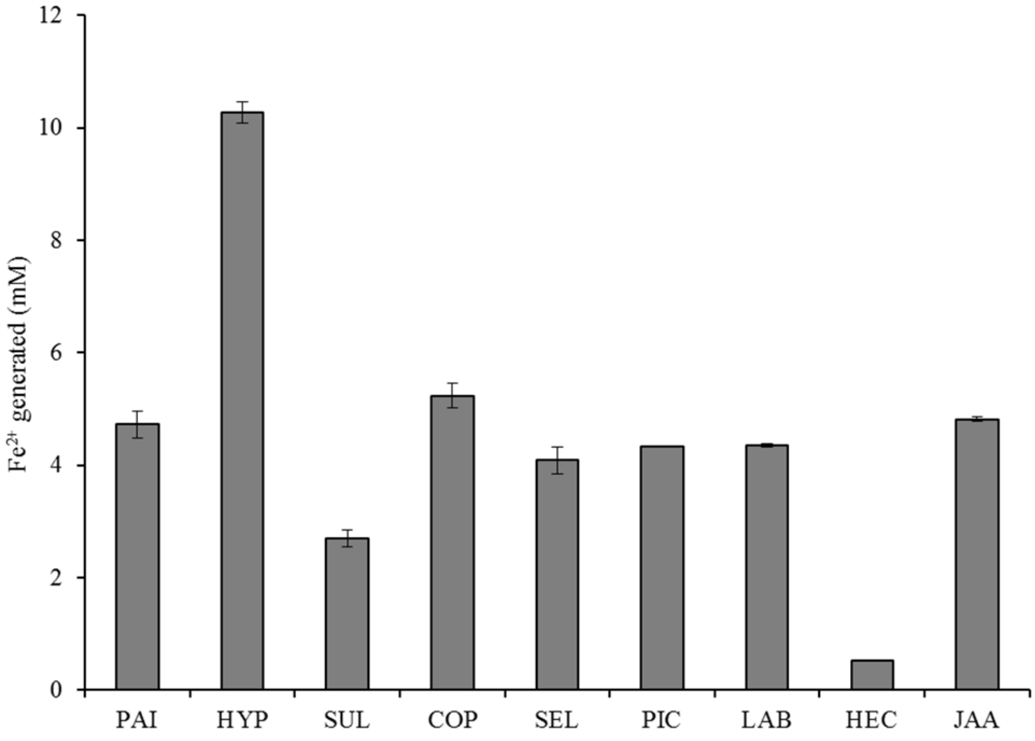

Fig. S5 PCA based on the expression patterns of orthologues. The expression analyses are based on the normalized expression levels from RNA-Seq data representing 3148 orthologues during growth on SOM (FH) and mineral nutrient medium (MMN) (all 3148 orthologues showed a false-discovery rate $q<0.01$). Each point in the PCA represents one species and one out of three replicates ($n=3$). The proportions of the variance explained by the principal components are indicated on each axis. The species abbreviations are listed in Table S1. In the top panel, the fungal species and their phylogeny are indicated. The bottom panel shows the same PCA as in the top panel, but the growth substrates are indicated.

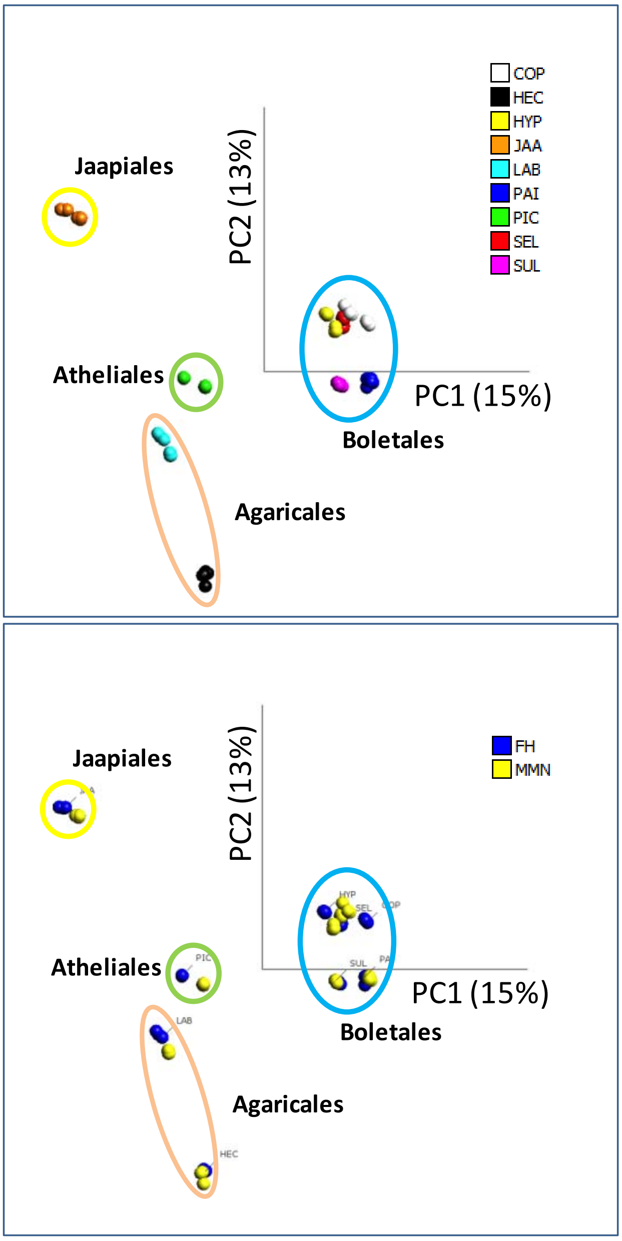

Fig. S6 Neighbour-joining gene expression trees. The gene expression trees are based on pairwise distance matrices between the fungi grown in SOM extract (FH) (*n*=3) *versus* mineral nutrient medium (MMN) (*n*=3). Sample distances between expression profiles were calculated as (**A**) Euclidian distances or (**B**) as (1 - *p*) where *p* is the Spearman's correlation coefficient. Bootstrap analysis was performed using 1000 replicates. Both trees were rooted with the *Jaapia argillacea* (JAA) FH and MMN samples. More details about the analysis in the main manuscript. The species abbreviations are listed in Table S1.

(A)

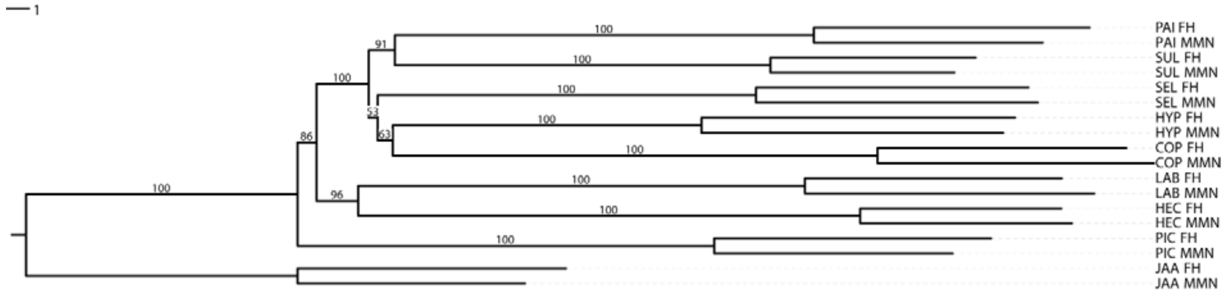

(B)

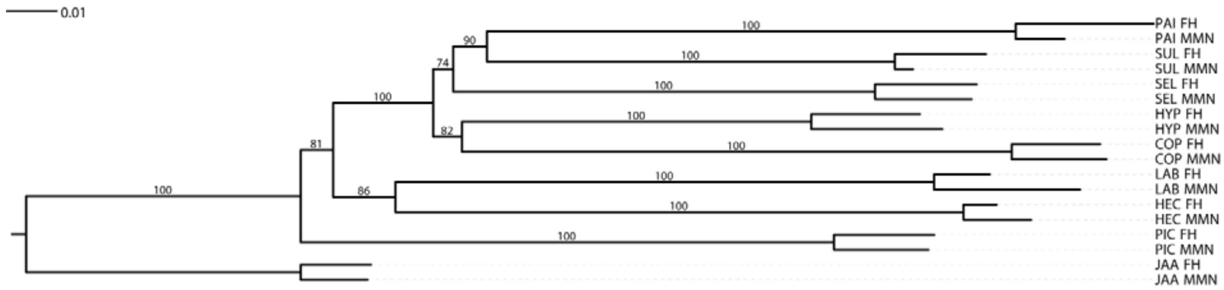

Fig. S7 Phylogenetic distribution of SOM-upregulated genes. The panel shows the expression and presence of SOM-upregulated genes in orthologous groups (rows, fold change >5 of pairwise comparisons in SOM extract *versus* MMN medium, q<0.01, n = 3) shared by at least two species (columns). The species abbreviations and clade affiliations (colour coded) are shown in the legend of Fig. 1. The size of the circles indicates the number of genes (if any) found in the orthologous groups within a given species and the black slice is proportional to the number of upregulated genes. The panels show orthologous group that were upregulated only in *C. puteana* (COP), *S. lacrymans* (SEL), *P. croceum* (PIC), *L. bicolor* (LAB), *H. cylindrosporum* (HEC) and *J. argillacea* (JAA). Orthologous groups that were upregulated in *P. involutus* (PAI), *H. pinastri* (HYP) and *S. luteus* (SUL), respectively and those upregulated in several species are shown in Fig. 3 (main manuscript). Annotations of the orthologue clusters are shown in Table S6.

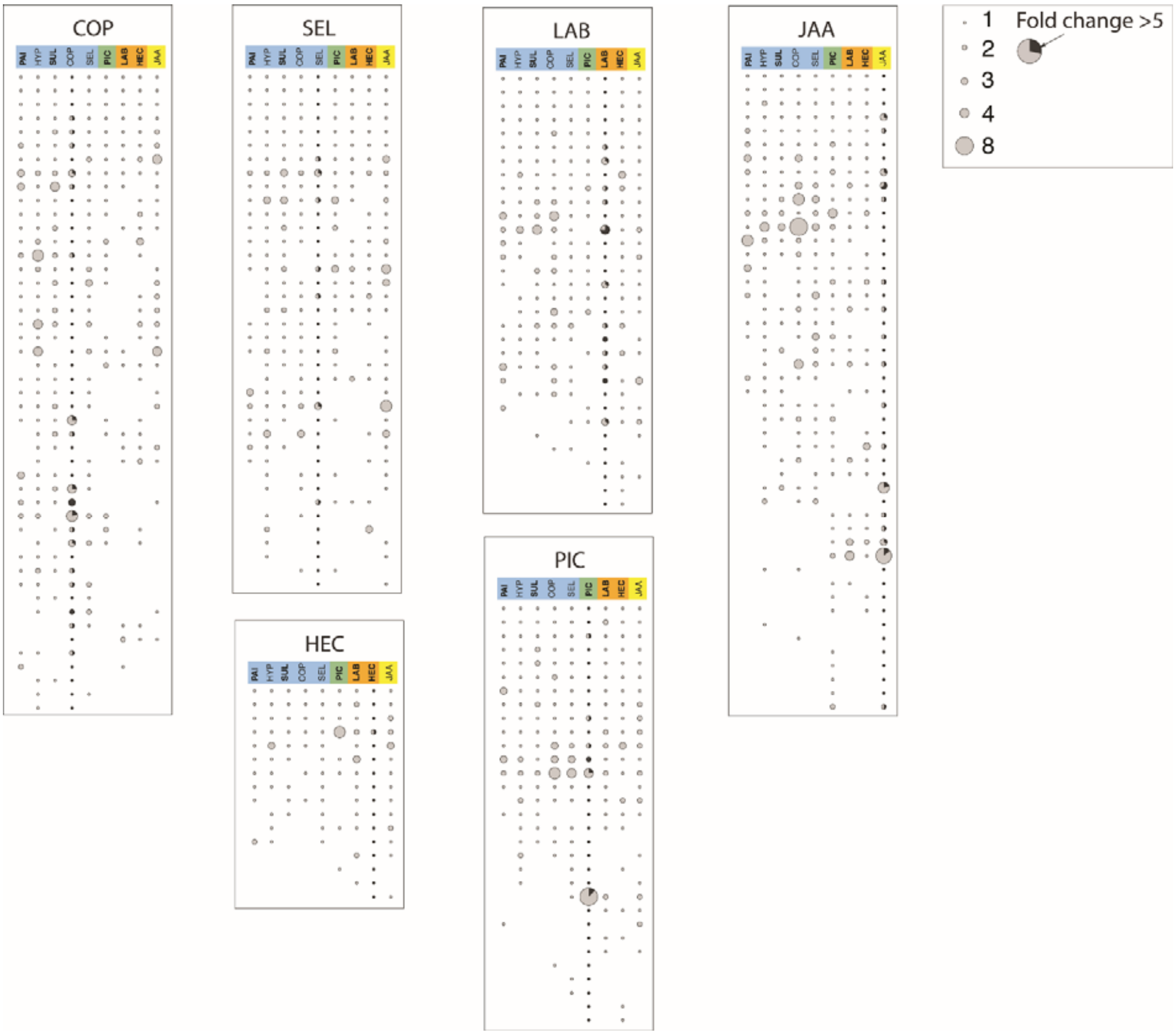

Fig. S8 Expression profiles of genes encoding auxiliary redox activities/enzymes (AAs). Shown is the average ratio of expression (*n*=3) of pairwise comparisons in media containing SOM extract *versus* mineral nutrient medium (MMN) as determined by RNA-Seq. Within each sub-panel, one for each species, the small boxes are representing individual gene models and the color shows the normalized fold change according to the color code below the panel. The species abbreviations are listed in Table S1. The gene models are listed in Table S8.

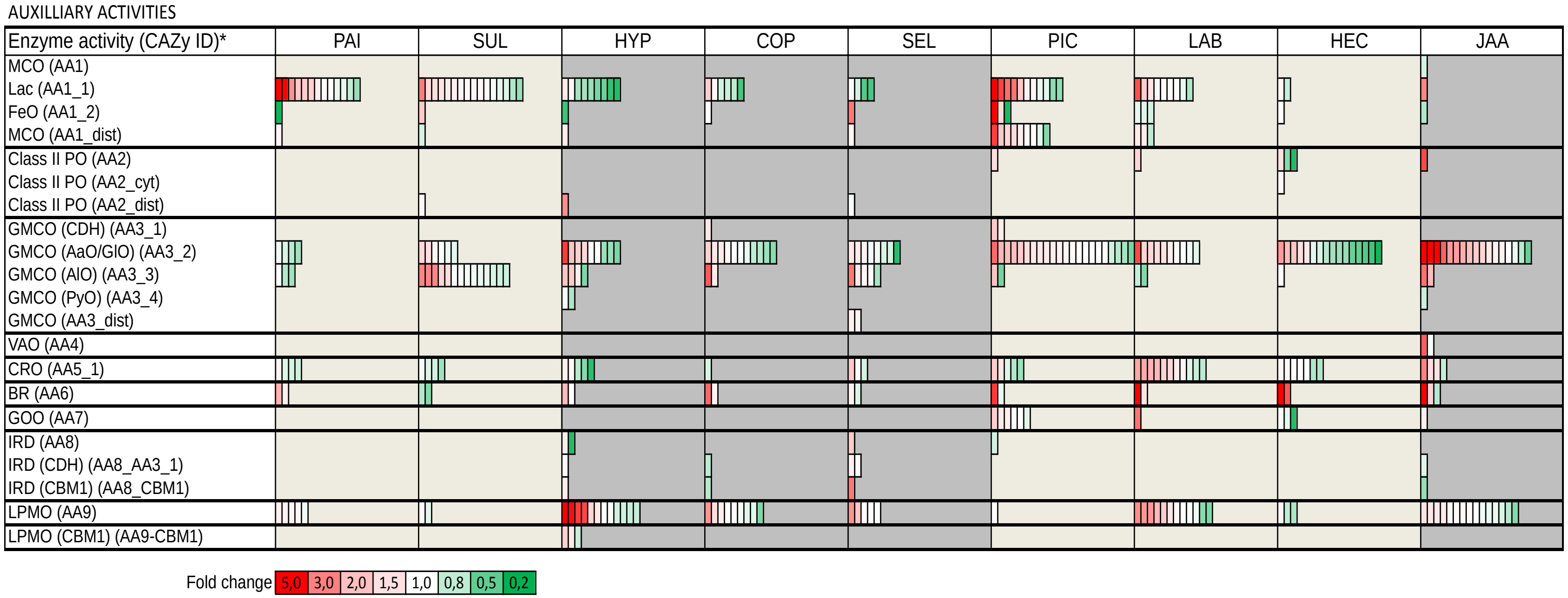

*MCO, Multicopper oxidase; Lac, Laccase; FeO, Ferroxidase; Class II PO, Class II Peroxidase; CDH, Cellobiose dehydrogenase; GMCO, GMC oxidoreductase; AaO, Aryl-alcohol oxidase; GlO, Glucose oxidase; AlO, Alcohol oxidase; PyO, Pyranose oxidase; VaO, Vanillyl-alcohol oxidase; CRO, copper-radical oxidase; BR, 1,4-Benzoquinone reductase; GOO, Glucooligosaccharide oxidase; IRD, Iron-reductase domain; LPMO, Lytic polysaccharide monooxygenase (formerly GH61); CBM1, Carbohydrate-binding module family 1 protein.

Fig. S9 Expression profiles of genes encoding peroxidases and tyrosinases. Shown is the average ratio of expression (*n*=3) of pairwise comparisons in media containing SOM extract of *versus* mineral nutrient medium (MMN) as determined by RNA-Seq. Within each sub-panel, one for each species, the small boxes are representing individual gene models and the color shows the normalized fold change according to the color code below the panel. The species abbreviations are listed in Table S1. The gene models are listed in Table S9.

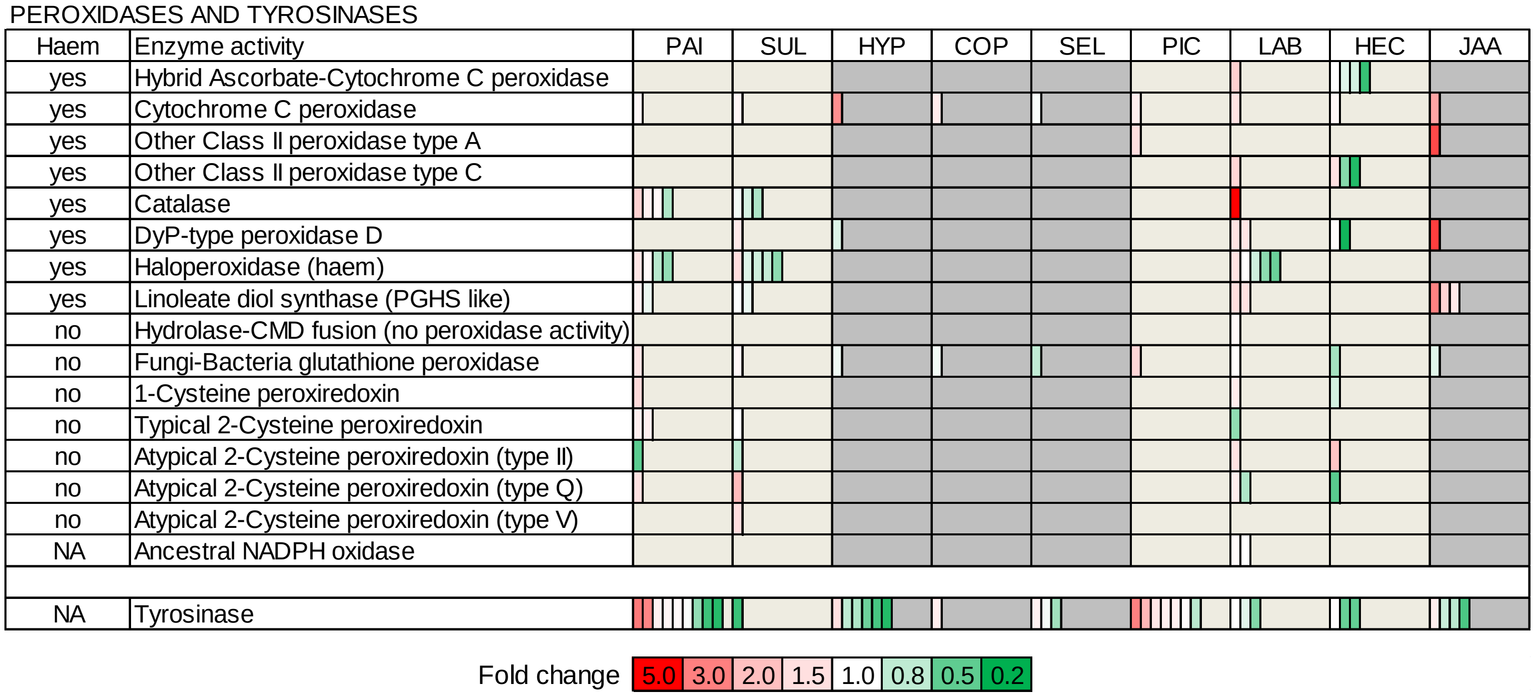

Fig. S10 Expression of genes encoding selected carbohydrate-modifying enzymes (CAZymes). Shown is the expression patterns of CAZymes expected to target cellulose, hemicelluloses. The species abbreviations are listed in Table S1. The gene models are listed in Table S10. **(A)** The average ratio of expression (*n*=3) of pairwise comparisons in media containing SOM extract *versus* mineral nutrient medium (MMN) as determined by RNA-Seq. Within each sub-panel, one for each species, the small boxes are representing individual gene models and the color shows the normalized fold change according to the color code below the panel.

(A)

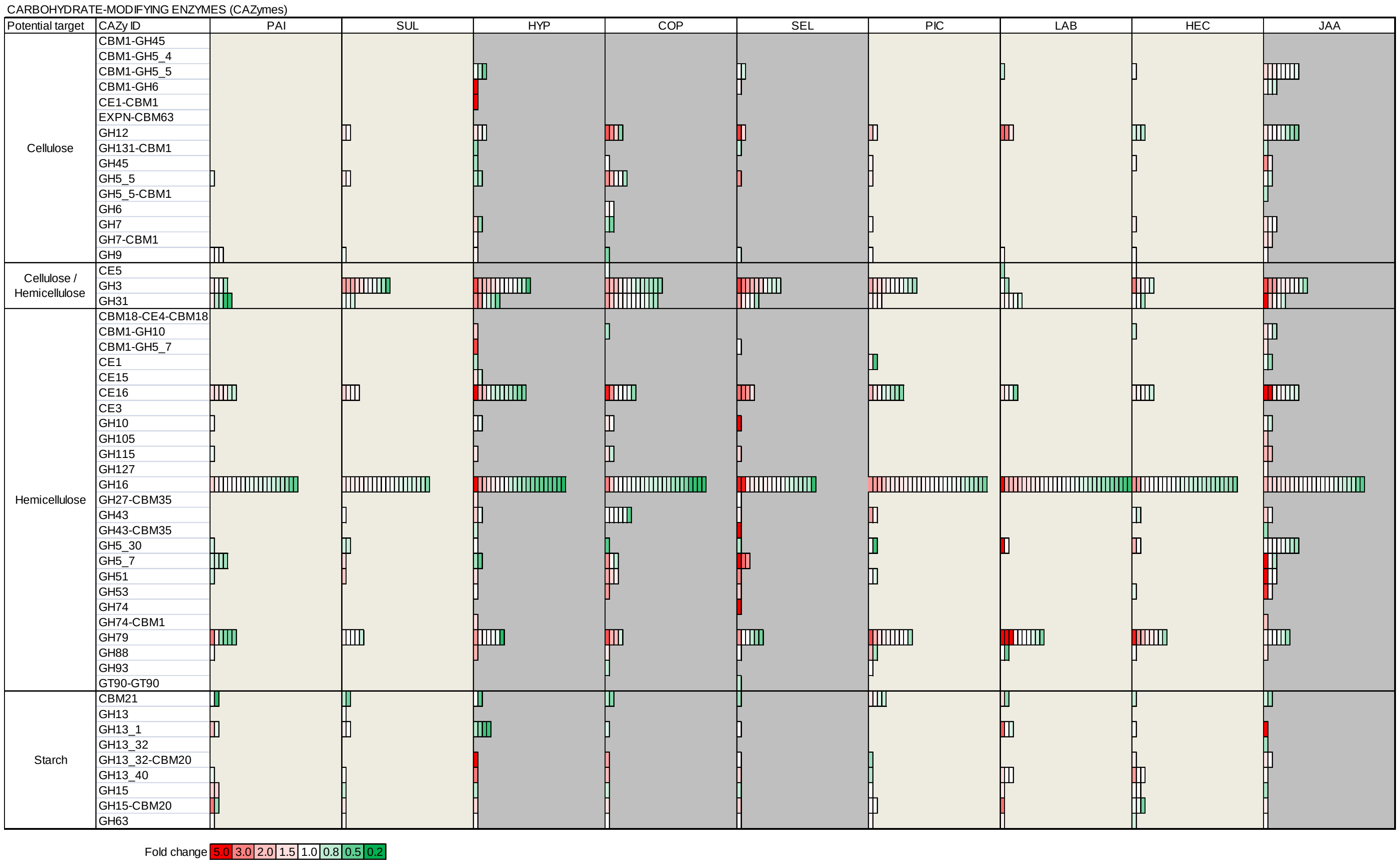

Fig. S10 (cont.) (B) The number of gene models found in the genomes (“Genome”), being SOM-upregulated (“SOM-Up (all)”) or being at least twofold SOM-upregulated (*q*<0.01, *n*=3, SOM *versus* MMN).

(B)

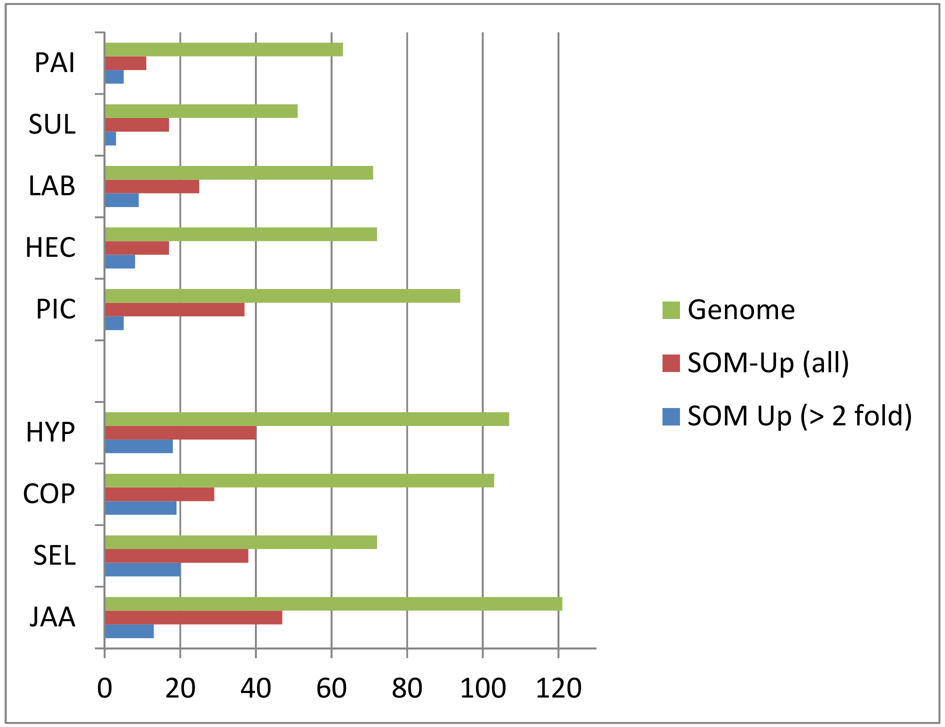

Fig. S11 Expression profiles of genes encoding extracellular peptidases. Shown is the average ratio of expression (*n*=3) of pairwise comparisons in SOM extract *versus* mineral nutrient medium (MMN). Within each sub-panel, one for each species, the small boxes are representing individual gene models and the color shows the normalized fold change according to the color code below the panel. The species abbreviations are listed in Table S1. Enzyme classes are according to MEROPS annotations (**Rawlings ND, Barrett AJ, Bateman A. 2012**. MEROPS: the database of proteolytic enzymes, their substrates and inhibitors. *Nucleic Acids Research* **40:** D343-D350). The gene models are listed in Table S11.

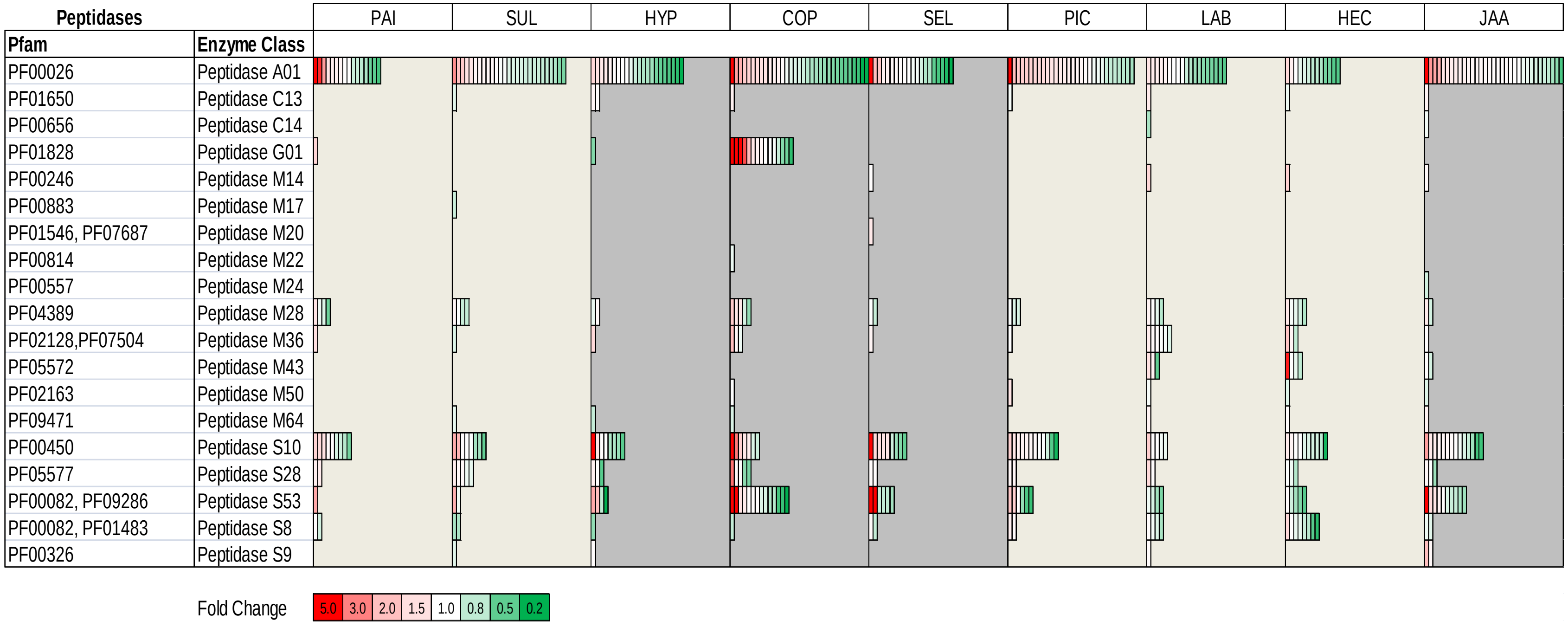

Fig. S12 Phylogeny and expression patterns of aspartate proteases. **(A)** Sequences from nine genomes were included (Table S1) and an unrooted phylogenetic tree of the Asp domain from 323 aspartate protease genes (Table S12) was reconstructed. Three genes from *H. pinastri* had identical amino-acid sequences in the Asp domain with other *H. pinastri* genes and were removed from the reconstruction (indicated with a star). Bootstrap values are shown for branches having >50 % support. Groups of genes labeled with P indicate paralog clades having more than three paralogs with >50% bootstrap support. Orthologues were labeled with O and the proteinOrtho group id. Significantly upregulated genes during growth on the SOM extract (FH) *versus* MMN media are shown with arrows. The tree can be viewed interactively online at: [http://itol.embl.de/external.cgi?tree=130235196501515141344627](http://itol.embl.de/external.cgi?tree=13023519650151514134462)70.

(A)

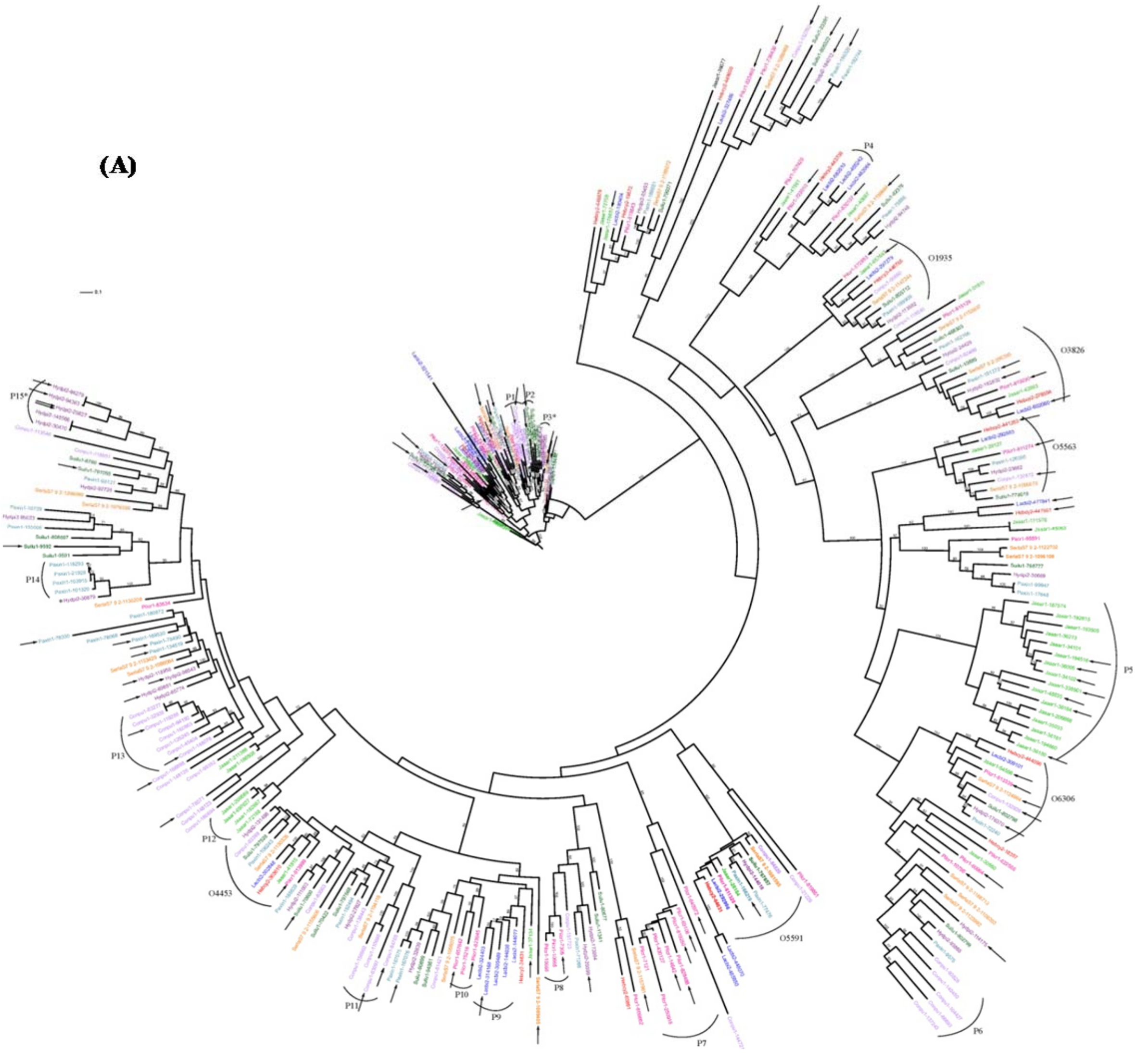

Fig. S12 (cont.) (B) Circos plot showing the proportion of 1:1 orthologues ("O"), paralogues ("P") and remaining genes ("R") of the aspartate protease family in the genome (right) and in the transcriptome during SOM decomposition (left). "O", "P" and "R" genes that were significantly SOM-upregulated (q<0.01, n=3, SOM extract *versus* MMN) are indicated in red, blue and green colors, respectively. Not upregulated "O", "P" and "R" genes are shown in grey. The species abbreviations are listed in Table S1.

(B)

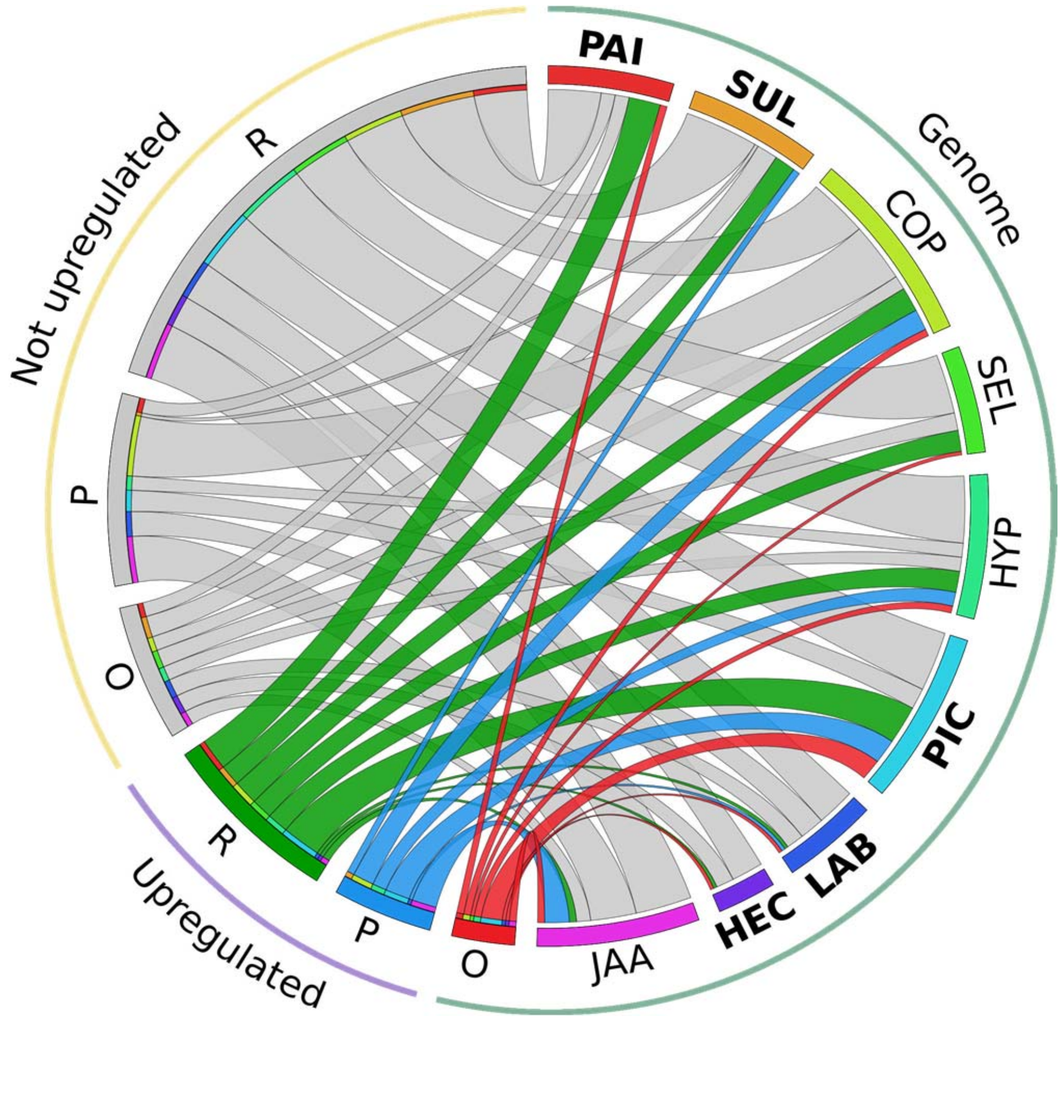

Fig. S13 Expression profiles of selected genes encoding biosynthetic enzymes involved in secondary metabolism. Shown is the average ratio of expression (*n*=3) of pairwise comparisons in SOM extract *versus* mineral nutrient medium (MMN). Within each sub-panel, one for each species, the small boxes are representing individual gene models and the color shows the normalized fold change according to the color code below the panel. The species abbreviations are listed in Table S1. The gene models are listed in Table S13.

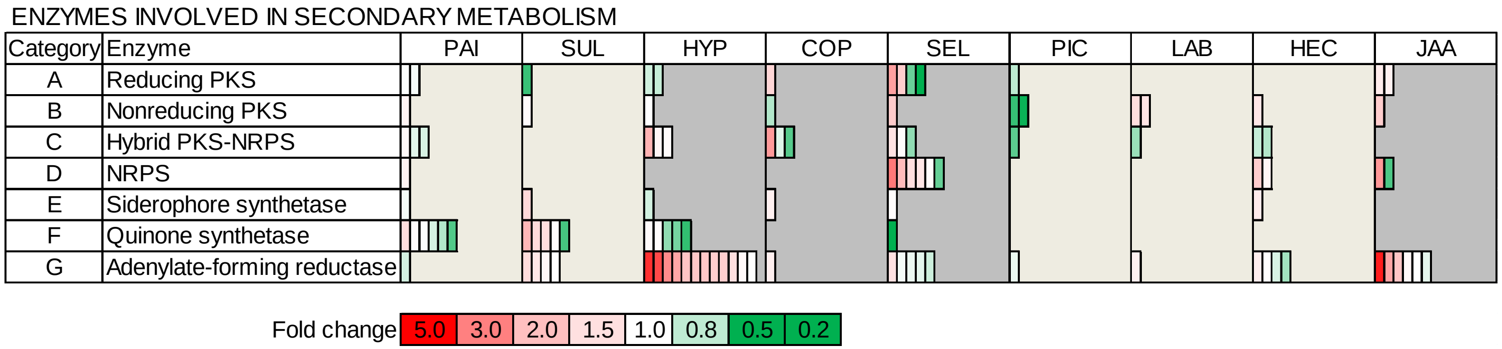

PKS, polyketide synthase; NRPS, Nonribosomal peptide synthetase.

Table S1 Fungi used in this study, and the assessment of the transcribed fraction of their genomes based on RNA-Seq data.

**Species**[a]	**Ecology**[b]	**Taxonomy**[c]	**Genome**[d]	**Transcribed**[e]			**Raw reads**[f]	**Mapped reads**[g]
				Total	Upregulated	Secreted	(10^6)	(10^6)
Paxillus involutus (PAI)	ECM (A)	Boletales	17968 (v1.0)	13912 (77%)	2174 (16%)	143 (6.6%)	260	170 (65%)
Hydnomerulius pinastri (HYP)	BR	Boletales	13270 (v2.0)	12101 (91%)	3467 (29%)	223 (6.4%)	228	143 (63%)
Suillus luteus (SUL)	ECM (A)	Boletales	18316 (v1.0)	15379 (84%)	2682 (17%)	159 (6.6%)	236	152 (64%)
Coniophora puteana (COP)	BR	Boletales	13761 (v1.0)	12229 (89%)	2808 (23%)	246 (8.8%)	252	167 (66%)
Serpula lacrymans (SEL)	BR	Boletales	12789 (v2.0)	10573 (83%)	2532 (24%)	196 (7.7%)	192	128 (67%)
Piloderma croceum (PIC)	ECM (B)	Atheliales	21583 (v1.0)	17901 (83%)	3584 (20%)	328 (9.2%)	188	127 (68%)
Laccaria bicolor (LAB)	ECM (C)	Agaricales	23130 (v2.0)	18886 (82%)	3261 (17%)	242 (6.6%)	223	151 (68%)
Hebeloma cylindrosporum (HEC)	ECM (D)	Agaricales	15382 (v2.0)	13958 (91%)	2471 (18%)	130 (5.3%)	281	150 (53%)
Jaapia argillacea (JAA)	WR-BR	Jaapiales	16419 (v1.0)	14741 (90%)	3989 (27%)	296 (7.4%)	301	219 (73%)

[a] The species-name abbreviations shown within parentheses are used throughout the manuscript. The following fungal strains were used in this study: *P. involutus* (ATCC 200175): *S. luteus* (UH-LM8N+N); *H. pinastri* (MO-312); *C. puteana* (Schum. Ex Fries, RWD-64-598SS-2); *S. lacrymans* (S7.9); *P. croceum* (DSMX-4824); *L. bicolor* ((Maire) P.D.Orton); *H. cylindrosporum* (Romagnesi TV98IV3); *J. argillacea* (MUCL-33604).

[b] ECM, ectomycorrhizal fungus; BR, brown-rot wood decayer; WR, white-rot wood decayer. Shown within parenthesis are the exploration types (A, long distance; B, short distance; C, medium-distance smooth subtype; D, short-or medium distance fringe subtype) (**Agerer R. 2001.** Exploration types of ectomycorrhizae. A proposal to classify ectomycorrhizal systems according to their patterns of differentiation and putative ecological importance. *Mycorrhiza* **11:** 107-114).

[c] Taxonomic affiliation are indicated by clade names.

[d] The number of predicted gene models and the version of the genome assembly (within parenthesis), as by the Joint Genome Institute (JGI) MycoCosm Portal (accessed 1 April 2015): *P. involutus* (http://genome.jgi-psf.org/Paxin1/Paxin1.home.html); *H. pinastri* (http://genome.jgi.doe.gov/Hydpi2/Hydpi2.home.html); *S. luteus* (http://genome.jgi-psf.org/Suilu1/Suilu1.home.html); *C. puteana* (http://genome.jgi.doe.gov/Conpu1/Conpu1.home.html); *S. lacrymans* (http://genome.jgi.doe.gov/SerlaS7_9_2/SerlaS7_9_2.home.html); *P. croceum* (http://genome.jgi-psf.org/Pilcr1/Pilcr1.home.html);

L. bicolor (http://genome.jgi-psf.org/Lacbi2/Lacbi2.home.html); *H. cylindrosporum* (http://genome.jgi.doe.gov/Hebcy2/Hebcy2.home.html)*; J. argillacea* (http://genome.jgi.doe.gov/Jaaar1/Jaaar1.home.html)

[e] The number of predicted gene models that were found to be transcribed in the present experiment. "Total" is the total number of gene models that were expressed (percentage of the total number of gene models); "Upregulated" is the number of genes that were significantly upregulated (q<0.01) in the medium containing the SOM extract *versus* mineral nutrient medium (percentage of the total number of expressed genes); "Secreted" is the number of the upregulated gene models that were predicted to contain a secretion signal (percentage of the total number of upregulated genes). Prediction of signal-peptide sequences was performed using SignalP 4.0.

[f] Number of raw reads by the RNA-Seq analysis.

[g] Number of unambiguously mapping reads that were used to compute the read statistics. Shown within parenthesis is the fraction as compared to the number of raw reads.

Table S2 Carbon and nitrogen concentrations of the SOM extract. “FH0” represents the initial extract before incubation with the fungus. Composition of the SOM extracts incubated for 7 days with various fungal strains are indicated by their abbreviated names; the species abbreviations are listed in Table S1. Concentrations are given in mg l^{-1} (mean ± SE, *n*=3). Abbreviations used: TOC, Total organic carbon; TN, Total nitrogen; Glucose-C, Glucose carbon.

	FH0	PAI	SUL	HYP	COP	SEL	PIC	LAB	HEC	JAA
TN	108 ± 0	37 ± 2	35 ± 0.3	49 ± 1	32 ± 1	60 ± 2	75 ± 1	70 ± 1	54 ± 0.7	55 ± 1
TOC	2324 ± 0	1258 ± 44	1138 ± 9	1628 ± 14	1347 ± 18	2050 ± 43	2238 ± 34	2005 ± 13	1715 ± 22	1710 ± 30
Glucose-C	1000 ±0	75 ± 67	70 ± 6	229 ± 32	306 ± 80	437 ± 198	556 ± 132	561 ± 16	427 ± 137	338 ± 123

Table S3 List of pyrolytic compounds identified by py-GC/MS analysis of the SOM extract.

Pyrolytic products	Origin	Retention time (min)	Fragment ions
Cyclopentanone	Aromatics	4.27	55
Hydroquinone, 2-methyl	Aromatics	11.92	124
Benzene	Benzene	2.75	78
Ethylbenzene	Benzene	5.23	91
1,2/3-Dimethylbenzene	Benzene	5.37	91
1,2(4)-Dimethylbenzene	Benzene	5.70	91
Benzofuran	Benzene	7.22	89
Dibenzofuran	Benzene	13.76	168
C16 Fatty acid	Fatty acids	17.59	73
Guaiacol	Lignins	8.47	109
4-methylguaiacol	Lignins	9.80	123
3-methoxycatechol	Lignins	10.75	140
ethylguaiacol	Lignins	10.85	137
Vinyl-Guaiacol	Lignins	11.31	150
Syringol	Lignins	11.72	154
Vanillin of iso-Vanillin of 4-Formylguaiacol	Lignins	12.39	151
trans-isoeugenol	Lignins	12.86	164
Acetylguaiacol	Lignins	13.33	151
Vanillic acid, methyl ester	Lignins	13.59	151
Guaiacylacetone	Lignins	13.74	180
Vanillic acid	Lignins	14.30	153
N-Nitrosodimethylamine	N-compounds	1.91	74
Propanenitrile, 2-methyl-	N-compounds	2.45	68
Pyrrole, 1-methyl-	N-compounds	3.55	80
Pyridine	N-compounds	3.88	52
Pyrrole	N-compounds	3.73	67
Acetamide	N-compounds	4.72	59
Pyridine, 2-methyl-	N-compounds	4.74	93
1H-Pyrrole, 2-methyl-	N-compounds	4.91	80
1H-Pyrrole, 3-methyl-	N-compounds	5.01	80
pyridine, 4-methyl	N-compounds	5.36	93
Pyridine, 2,6-dimethyl-	N-compounds	5.62	107
4(1H)-Pyrimidinone, 6-methyl-	N-compounds	7.04	110
2-Propanamine, N-methyl-N-nitroso-	N-compounds	9.24	102
Indole	N-compounds	11.17	90
Thymine	N-compounds	15.54	126
Diketodipyrrole	N-compounds	15.62	186

Indene	Polyaromatics	7.91	116
x-Methyl naphthalene	Polyaromatics	11.23	124
Phenol	Phenols	6.96	66
2-Methylphenol	Phenols	7.98	108+107
4-methylphenol	Phenols	8.30	108+107
Phenol, 2-ethyl	Phenols	9.08	107
Phenol, 2,4-dimethyl	Phenols	9.24	107
Phenol, 4-ethyl	Phenols	9.48	107
phenol, 4-vinyl	Phenols	10.14	120
2-methylfuran	Polysaccharides	2.26	82
Acetic acid	Polysaccharides	2.95	60
Propanoic acid, methyl ester	Polysaccharides	2.48	57
1,3-Cyclopentadiene, 1-methyl- + 1,3-Cyclohexadiene	Polysaccharides	2.56	80
2-Butenal (E)	Polysaccharides	2.66	70
2-Propanone, 1-hydroxy-	Polysaccharides	3.12	74
Furan, 2-ethyl+dimethyl	Polysaccharides	3.16	81
Propanoic acid	Polysaccharides	3.64	74
Propanoic acid, 2-oxo-, methyl ester	Polysaccharides	4.35	102
Furan, 2-ethyl-5-methyl-	Polysaccharides	4.33	110
(2H)-Furan-3-one	Polysaccharides	4.51	55
Furan, 2,3,5-trimethyl-	Polysaccharides	4.53	110
3-Furaldehyde	Polysaccharides	4.59	96
2,5-Furandione	Polysaccharides	4.85	98
Furfural	Polysaccharides	4.80	95
2-Cyclopenten-1-one	Polysaccharides	4.88	82
2-Cyclopenten-1,4-dione	Polysaccharides	4.92	42
2-Furanmethanol	Polysaccharides	5.20	98
Furan, 2-(2-propenyl)-	Polysaccharides	5.12	108
2-Propanone, 1-(acetyloxy)-	Polysaccharides	5.36	86
2-propylfuran	Polysaccharides	5.29	110
2(3H)-Furanone, 5-methyl-	Polysaccharides	5.32	55
2-Cyclopentene-1,4-dione	Polysaccharides	5.54	42
styrene	Polysaccharides	5.68	104
2-Cyclopenten-1-one, 2-methyl-	Polysaccharides	5.87	67
Ethanone, 1-(2-furanyl)-	Polysaccharides	5.93	95
2(5H)-Furanone	Polysaccharides	6.03	55
2-Cyclopenten-1-one, 2-hydroxy-	Polysaccharides	6.22	98
2(5H)-Furanone, 5-methyl-	Polysaccharides	6.38	55
2-Furancarboxaldehyde, 5-methyl-	Polysaccharides	6.68	109
2-Cyclopenten-1-one, 3-methyl-	Polysaccharides	6.77	96
(2H)-Pyran-2-one, 4-hydroxy, 5,6-dihydro-	Polysaccharides	7.32	114

3-hydroxy-2-methyl-2-cyclopenten-1-one	Polysaccharides	7.57	112
2-hydroxy-3-methyl-2-cyclopenten-1-one	Polysaccharides	7.67	112
2-Cyclopenten-1-one, 2,3-dimethyl-	Polysaccharides	7.79	67
Dianhydrorhamnose	Polysaccharides	7.94	113
3-hydroxy-2-methyl-(4H)-pyran-4-one (maltol)	Polysaccharides	8.85	126
4H-Pyran-4-one, 2,3-dihydro-3,5-dihydroxy-6-methyl-	Polysaccharides	9.37	144
4H-Pyran-4-one, 3,5-dihydroxy-x-methyl-	Polysaccharides	9.73	142
4H-Pyran-4-one, 3,5-dihydroxy-x-methyl-	Polysaccharides	9.93	142
Xanthosine	Polysaccharides	10.45	73
1,4:3,6-Dianhydro-à-d-glucopyranose	Polysaccharides	10.33	69
2-Furancarboxaldehyde, 5-(hydroxymethyl)-	Polysaccharides	10.55	126
1H-Inden-1-one, 2,3-dihydro-	Polysaccharides	11.03	104
1,4-Dideoxy-D-glycero-hex-1+	Polysaccharides	11.71	144
Levogalactosan	Polysaccharides	13.07	60
Levomannosan	Polysaccharides	13.86	60
Levoglucosan	Polysaccharides	14.69	60
Catechol	Tannins	9.95	110
Catechol, 4-methyl	Tannins	10.73	124
Toluene	Toluenes	3.93	91
2-methoxytoluene	Toluenes	7.53	122

Table S4 Numbers of gene models in various (co)-orthologous groups. Numbers in grey boxes represent 1:1 orthologues. The species abbreviations are listed in Table S1.

	**Orthologues and species specific genes**									**Co-orthologues**								
	**PAI**	**HYP**	**SUL**	**COP**	**SEL**	**PIC**	**LAB**	**HEC**	**JAA**	**PAI**	**HYP**	**SUL**	**COP**	**SEL**	**PIC**	**LAB**	**HEC**	**JAA**
**Genome**																		
Species specific	9339	4706	10108	6518	5043	13432	15126	7847	8681	0	0	0	0	0	0	0	0	0
Orthologues (2 species)	713	629	469	242	331	536	683	598	333	53	31	21	13	11	26	35	33	14
Orthologues (3 species)	311	330	293	163	249	288	241	221	211	155	135	125	67	71	157	179	120	130
Orthologues (4 species)	238	283	222	168	244	234	179	173	219	253	186	206	167	156	210	196	136	200
Orthologues (5 species)	219	266	232	192	261	206	124	129	186	304	251	219	226	227	219	221	170	209
Orthologues (6 species)	235	266	246	190	235	197	162	161	204	304	301	313	290	240	269	246	208	268
Orthologues (7 species)	332	370	368	260	351	351	289	285	320	250	257	225	256	237	253	196	157	233
Orthologues (8 species)	756	819	819	554	795	796	780	774	787	379	372	372	346	333	348	330	334	365
Orthologues (9 species)	3148	3148	3148	3148	3148	3148	3148	3148	3148	979	920	930	961	857	913	995	888	911
1:1 Orthologues (%)	17.5	23.7	17.2	22.9	24.6	14.6	13.6	20.5	19.2									
**Transcribed**																		
Species specific	5993	3892	7888	5321	3229	10313	11324	6748	7339	0	0	0	0	0	0	0	0	0
Orthologues (2 species)	532	545	291	191	240	412	593	535	276	28	18	7	8	6	20	31	29	10
Orthologues (3 species)	240	301	229	143	201	229	207	196	180	111	113	77	50	51	116	133	99	111
Orthologues (4 species)	202	253	176	146	218	207	153	156	206	168	144	122	121	110	161	157	111	164
Orthologues (5 species)	202	258	213	180	247	195	113	118	173	206	205	150	182	176	136	150	127	156
Orthologues (6 species)	229	261	241	186	232	191	157	158	201	239	269	216	247	208	204	197	152	215
Orthologues (7 species)	330	367	365	259	347	347	285	283	317	201	230	169	223	205	198	162	125	202
Orthologues (8 species)	756	819	817	553	793	796	780	774	786	353	361	346	326	318	325	310	319	350
Orthologues (9 species)	3148	3148	3148	3148	3148	3148	3148	3148	3148	974	917	924	947	845	903	986	880	907
1:1 Orthologues (%)	22.6	26.0	20.5	25.7	29.8	17.6	16.7	22.6	21.4									

Table S4 (cont.)

	Orthologues and species specific genes									Co-orthologues								
	PAI	HYP	SUL	COP	SEL	PIC	LAB	HEC	JAA	PAI	HYP	SUL	COP	SEL	PIC	LAB	HEC	JAA
**Upregulated**																		
Species specific	560	792	806	1074	507	1294	1027	947	1316	0	0	0	0	0	0	0	0	0
Orthologues (2 species)	73	151	40	51	43	81	118	94	59	1	4	4	1	1	8	4	12	2
Orthologues (3 species)	61	106	54	51	56	56	40	29	61	23	19	21	16	11	25	24	22	32
Orthologues (4 species)	46	78	47	45	56	49	38	28	54	25	30	37	37	32	32	25	20	47
Orthologues (5 species)	39	80	45	42	76	59	30	25	50	43	49	42	65	56	39	25	25	33
Orthologues (6 species)	38	81	66	56	67	55	46	31	85	52	71	77	91	63	40	43	36	61
Orthologues (7 species)	61	110	95	61	96	121	78	63	104	35	67	59	80	61	49	45	35	68
Orthologues (8 species)	144	267	200	125	228	242	219	149	306	84	113	117	120	112	110	100	72	137
Orthologues (9 species)	637	1100	654	635	822	1004	1047	646	1195	252	349	318	258	245	320	352	237	379
1:1 Orthologues (%)	29.3	31.7	24.4	22.6	32.5	28.0	32.1	26.1	30.0									
**Upregulated, Secretome**																		
Species specific	44	86	59	95	37	138	99	74	111	0	0	0	0	0	0	0	0	0
Orthologues (2 species)	7	7	0	3	1	8	13	7	3	1	0	0	1	1	4	1	0	0
Orthologues (3 species)	1	10	1	11	12	5	8	1	14	1	2	0	2	2	6	5	4	2
Orthologues (4 species)	4	7	2	6	9	8	5	1	5	2	9	3	8	3	5	6	2	12
Orthologues (5 species)	2	7	2	6	5	5	3	1	4	5	7	5	9	8	9	4	3	5
Orthologues (6 species)	1	6	4	1	2	5	5	1	5	11	10	11	23	13	7	14	4	6
Orthologues (7 species)	3	2	3	2	8	8	4	1	7	6	10	5	9	7	12	8	4	11
Orthologues (8 species)	3	6	6	8	9	7	5	1	15	10	10	15	21	19	11	10	3	17
Orthologues (9 species)	27	25	18	20	36	52	28	11	43	15	19	25	21	24	38	24	12	36
1:1 Orthologues (%)	18.9	11.2	11.3	8.1	18.4	15.9	11.6	8.5	14.5									

Table S5 Numbers of highly SOM-upregulated genes (fold change >5 of pairwise comparisons in SOM extract *versus* MMN medium, q<0.01, n = 3). The species abbreviations are listed in Table S1.

	**PAI**	**HYP**	**SUL**	**COP**	**SEL**	**PIC**	**LAB**	**HEC**	**JAA**	(Sum)
Total	35	96	18	135	91	85	86	56	113	(715)
Orthologues	30	51	14	69	64	34	45	24	56	(387)
Species specific	5	45	4	66	27	51	41	32	57	(328)

Table S6 Annotation of highly SOM-upregulated orthologues. The table shows the annotation of 324 orthologue clusters containing genes that were at least 5-fold SOM-upregulated (pairwise comparisons in SOM extract *versus* MMN medium, $q<0.01$, $n = 3$) (*c.f.* Fig. 3, main manuscript).

Ortho Id	Pfam	Pfam name	CAZymes and AAs[a]	MEROPS[b]	Secondary metab.[c]	Secreted[d]
**4 species**						
4675	PF00704,PF02839	Glyco_hydro_18,CBM_5_12				Y
934	PF00083	Sugar_tr				
**3 species**						
5484	PF02055	Glyco_hydro_30				Y
5745	PF00011	HSP20				
5008	PF03746	LamB_YcsF				
2839	PF07690	MFS_1				
632	PF03169	OPT				
5340	PF03358,PF00258	FMN_red,Flavodoxin_1	AA6			
**2 species**						
6039	PF00491	Arginase				Y
6107	PF07732,PF00394, PF07731	Cu-oxidase_3,Cu-oxidase,Cu-oxidase_2	AA1_1			Y
616	PF07690,PF00366	MFS_1,Ribosomal_S17				
1647	PF11790	Glyco_hydro_cc				Y
3462	PF01786	AOX				
2470	PF00389,PF02826	2-Hacid_dh,2-Hacid_dh_C				
5838	PF00657	Lipase_GDSL	CE16			Y
4563	PF01408	GFO_IDH_MocA				
273	PF02839,PF00704	CBM_5_12,Glyco_hydro_18				Y
741	PF00026	Asp		A01		Y
5056	PF13632	Glyco_trans_2_3				
1507						
4888	PF02133	Transp_cyt_pur				
5711	PF09286,PF00082	Pro-kuma_activ,Peptidase_S8		S53		Y
1697	PF01187	MIF				
5924	PF00171	Aldedh				
3914	PF02839	CBM_5_12				Y
3965	PF09286,PF00082	Pro-kuma_activ,Peptidase_S8		S53		Y
1047	PF00174,PF03404, PF00173,PF00970, PF00175	Oxidored_molyb,Mo-co_dimer,Cyt-b5,FAD_binding_6,NAD_binding_1				
3487	PF02353	CMAS				
2639						
3476	PF00011	HSP20				

Ortho Id	Pfam	Pfam name	CAZymes and AAs[a]	MEROPS[b]	Secondary metab.[c]	Secreted[d]
2062	PF00150	Cellulase	GH5_7,GH5_30			Y
1001	PF00248	Aldo_ket_red				
6552	PF01185	Hydrophobin				Y
4349	PF03936	Terpene_synth_C				
5065	PF01370	Epimerase				
891	PF01545	Cation_efflux				
**PAI**						
2032						
3396	PF06127	DUF962				
3450	PF00463	ICL				
4240	PF13561,PF00106	adh_short_C2,adh_short				
5641	PF03009	GDPD				
3615	PF00999	Na_H_Exchanger				
282	PF00125	Histone				
1	PF00656	Peptidase_C14				
1074	PF07690	MFS_1				
6673	PF00149	Metallophos				Y
6958						
5623	PF00651	BTB				
6752	PF04143,PF14241	Sulf_transp,DUF4341				
3877	PF00501,PF07993, PF00550	AMP-binding,NAD_binding_4,PP-binding			Adenylate-forming reductase	
2752	PF04140,PF04191	ICMT,PEMT				
5328	PF01185	Hydrophobin				Y
5116	PF14027	DUF4243				
7023	PF07714	Pkinase_Tyr				
1756	PF00026	Asp		A01		Y
9563	PF13391	HNH_2				
9457	PF02055	Glyco_hydro_30				Y
7625						
**HYP**						
521	PF02861,PF00004, PF07724,PF10431, PF10276	Clp_N,AAA,AAA_2,ClpB_D2-small,zf-CHCC				
2683						Y
4216	PF07992,PF00070	Pyr_redox_2,Pyr_redox				
4313	PF00011	HSP20				
779	PF13673,PF00583, PF08445	Acetyltransf_10,Acetyltransf_1,FR47				

Ortho Id	Pfam	Pfam name	CAZymes and AAs[a]	MEROPS[b]	Secondary metab.[c]	Secreted[d]
3784	PF00854	PTR2				
5917	PF08241	Methyltransf_11				
3770	PF00728,PF02838	Glyco_hydro_20,Glyco_hydro_20b				Y
5349	PF13668	Ferritin_2				Y
5925	PF03798	TRAM_LAG1_CLN8				
1429	PF00069	Pkinase				
4098	PF01408,PF02894	GFO_IDH_MocA,GFO_IDH_MocA_C				
2734						
8267	PF13847,PF08241,PF13489	Methyltransf_31,Methyltransf_11,Methyltransf_23				
4848	PF11937	DUF3455				Y
1261	PF03330	DPBB_1				Y
7035						
4157	PF00106	adh_short				
2650	PF00248	Aldo_ket_red				
7226	PF00035	dsrm				
7688	PF01425	Amidase				
7895	PF00106,PF13561	adh_short,adh_short_C2				
7721	PF00722	Glyco_hydro_16	GH16			Y
5329	PF10022	DUF2264				
4988	PF00128,PF02806,PF00686	Alpha-amylase,Alpha-amylase_C,CBM_20				Y
3564	PF06985	HET				
2621	PF13489,PF08241	Methyltransf_23,Methyltransf_11				
7628						Y
7147	PF00646,PF12937	F-box,F-box-like				
8067	PF13561	adh_short_C2				
1152	PF01341,PF00734	Glyco_hydro_6,CBM_1	GH6,CBM1-GH6			Y
4866	PF03443	Glyco_hydro_61	AA9			Y
1810	PF01183	Glyco_hydro_25				Y
8082						
3425						
2073	PF00083	Sugar_tr				
7612						
7883						
7478	PF00450	Peptidase_S10		S10		Y
8560						

Ortho Id	Pfam	Pfam name	CAZymes and AAs[a]	MEROPS[b]	Secondary metab.[c]	Secreted[d]
8083						
4122						
**SUL**						
5280	PF09729	Gti1_Pac2				
5313	PF00701	DHDPS				
5523	PF03992	ABM				
4292	PF01168,PF14031	Ala_racemase_N,D-ser_dehydrat				
692						
2901	PF00083	Sugar_tr				
546	PF07991	IlvN				
547	PF00106,PF13561	adh_short,adh_short_C2				
543	PF07859	Abhydrolase_3				
10271	PF00248	Aldo_ket_red				
**COP**						
1880	PF02560	Cyanate_lyase				
4215	PF00568,PF00786	WH1,PBD				
4738	PF00248	Aldo_ket_red				
1834	PF06027	DUF914				
2354	PF00117	GATase				
5289	PF01425	Amidase				
4641	PF13417,PF13410, PF00043	GST_N_3,GST_C_2,GST_C				
1369	PF03169	OPT				
6004	PF08450	SGL				Y
2419	PF00150	Cellulase				
3903	PF13714	PEP_mutase				
485	PF01425	Amidase				
4765	PF00106	adh_short				
5825	PF00106	adh_short				
1522	PF03169	OPT				
4843	PF05368	NmrA				
6447	PF01425	Amidase				
6037	PF01522	Polysacc_deac_1				
4566	PF00753,PF12706	Lactamase_B,Lactamase_B_2				
4973	PF05378,PF01968, PF06032	Hydant_A_N,Hydantoinase_A, DUF917				
6261	PF13460	NAD_binding_10				Y
3894	PF06687	SUR7				
3902	PF00173,PF01070	Cyt-b5,FMN_dh				
4606	PF01546,PF07687	Peptidase_M20,M20_dimer		M20		

**Ortho Id**	**Pfam**	**Pfam name**	**CAZymes and AAs[a]**	**MEROPS[b]**	**Secondary metab.[c]**	**Secreted[d]**
5302	PF01425	Amidase				
2710	PF00270,PF00271	DEAD,Helicase_C				
2229	PF01593,PF13450	Amino_oxidase,NAD_binding_8				
5796	PF00474	SSF				
3718	PF03372	Exo_endo_phos				Y
3474	PF13520	AA_permease_2				
5807	PF00324	AA_permease				
8	PF01185	Hydrophobin				Y
5823	PF00106	adh_short				
4413	PF11807	DUF3328				
1083	PF13450,PF00743	NAD_binding_8,FMO-like				
4173						
3819						
2489	PF00732,PF05199	GMC_oxred_N,GMC_oxred_C	AA3_3			
4184						
5104	PF14226,PF03171, PF10489	DIOX_N,2OG-FeII_Oxy,RFPL3_antisense				
1038						
5094						Y
2928	PF01828	Peptidase_A4		G01		Y
2789	PF00067	p450				
702	PF00491	Arginase				
4090	PF00474	SSF				
5303						
**SEL**						
1777	PF04199	Cyclase				
3824	PF00782	DSPc				
3951	PF13847,PF00581	Methyltransf_31,Rhodanese				
4070	PF00857	Isochorismatase				
5370	PF08609,PF11698	Fes1,V-ATPase_H_C				
6040	PF01554	MatE				
2155	PF06283	ThuA				
3842	PF01738	DLH				
1881	PF07350	DUF1479				
5533	PF00171	Aldedh				
3017	PF00875,PF03441	DNA_photolyase,FAD_binding_7				
5670	PF09248,PF01179	DUF1965,Cu_amine_oxid				
3662	PF00328	His_Phos_2				
8368	PF04191,PF04140	PEMT,ICMT				

Ortho Id	Pfam	Pfam name	CAZymes and AAs[a]	MEROPS[b]	Secondary metab.[c]	Secreted[d]
7476	PF12697	Abhydrolase_6				
2544						Y
4147	PF00450	Peptidase_S10		S10		Y
4440	PF07690	MFS_1				
5894	PF12697,PF00561	Abhydrolase_6,Abhydrolase_1				
541	PF00857	Isochorismatase				
4839	PF00248	Aldo_ket_red				
1911	PF03069	FmdA_AmdA				
1220						Y
1060	PF03928	DUF336				
6379	PF00248	Aldo_ket_red				
6914						
1085	PF00331,PF00734	Glyco_hydro_10,CBM_1	GH10,CBM1-GH10			Y
8271	PF00106	adh_short				
8190	PF00704,PF02839	Glyco_hydro_18,CBM_5_12				Y
6377	PF13823,PF08240, PF00107	ADH_N_assoc,ADH_N,ADH_zinc_N				
2056						
9365	PF00450	Peptidase_S10		S10		Y
5193	PF00232	Glyco_hydro_1				
7054	PF01185	Hydrophobin				Y
6830	PF00734	CBM_1	GH74-CBM1,GH74			Y
6921	PF04616,PF03422	Glyco_hydro_43,CBM_6	GH43-CBM35			
4386	PF04140	ICMT				
10852	PF01370	Epimerase				
**PIC**						
1490	PF02678,PF05726	Pirin,Pirin_C				
209	PF00909	Ammonium_transp				
923	PF02668	TauD				
1169	PF01699	Na_Ca_ex				
1667	PF01144	CoA_trans				
2185	PF00230	MIP				
1795	PF04444,PF00775	Dioxygenase_N,Dioxygenase_C				
2445	PF01274	Malate_synthase				
2884	PF01328	Peroxidase_2				
3831	PF00083	Sugar_tr				
1903						
4047	PF00891	Methyltransf_2				

Ortho Id	Pfam	Pfam name	CAZymes and AAs[a]	MEROPS[b]	Secondary metab.[c]	Secreted[d]
1820	PF00067	p450				
1426	PF00403	HMA				
6402	PF14200	RicinB_lectin_2				
7698	PF04749	PLAC8				
4727						
2922	PF00026	Asp		A01		Y
2188	PF07690	MFS_1				
7150	PF00226,PF01556	DnaJ,DnaJ_C				
7600	PF01261	AP_endonuc_2				
9592	PF00891	Methyltransf_2				
9793	PF02812,PF00208	ELFV_dehydrog_N,ELFV_dehydrog				
10087	PF01494	FAD_binding_3				
9660	PF00127	Copper-bind				Y
9517						Y
4014						Y
10523						
10709	PF02666	PS_Dcarbxylase				
10331						Y
10620	PF00083	Sugar_tr				
**LAB**						
1621	PF03060	NMO				
4025	PF00929	RNase_T				
5276	PF08240,PF00107	ADH_N,ADH_zinc_N				
5987	PF04588	HIG_1_N				
4394	PF00199,PF06628	Catalase,Catalase-rel				
5173	PF00083	Sugar_tr				
4761	PF00690,PF00122,PF00702	Cation_ATPase_N,E1-E2_ATPase,Hydrolase				
790	PF07992,PF00070	Pyr_redox_2,Pyr_redox				
5814	PF01184	Grp1_Fun34_YaaH				
6078	PF00150	Cellulase				Y
4758	PF07690	MFS_1				
2305	PF13417,PF00043,PF02798	GST_N_3,GST_C,GST_N				
3966	PF00248	Aldo_ket_red				
3697	PF00107	ADH_zinc_N				
6	PF01040	UbiA				
2527	PF01583	APS_kinase				
2721	PF12051	DUF3533				
84	PF00266	Aminotran_5				

Ortho Id	Pfam	Pfam name	CAZymes and AAs[a]	MEROPS[b]	Secondary metab.[c]	Secreted[d]
3494	PF01764	Lipase_3				Y
5510			GH79			Y
6915						Y
2581	PF06330	TRI5				
111	PF01185	Hydrophobin				Y
5327	PF02798,PF00043, PF13417	GST_N,GST_C,GST_N_3				
9403	PF00067	p450				
8651	PF03351,PF03188	DOMON,Cytochrom_B561				Y
8672	PF00854	PTR2				
4329						
8983	PF12296	HsbA				Y
8679	PF07690	MFS_1				
8701	PF00324	AA_permease				
9620						
**HEC**						
3942	PF08031,PF01565	BBE,FAD_binding_4				
902	PF02727,PF02728, PF01179	Cu_amine_oxidN2,Cu_amine_ oxidN3,Cu_amine_oxid				
4751	PF00083	Sugar_tr				
4831	PF00067	p450				
6354	PF13417,PF13410	GST_N_3,GST_C_2				
7428	PF02678	Pirin				
2685	PF08450,PF01731	SGL,Arylesterase				
7679	PF00498	FHA				
1260	PF03330	DPBB_1				Y
7604	PF00172	Zn_clus				
7044						
7853	PF07883,PF00190	Cupin_2,Cupin_1				Y
8897						
10573						
9807	PF01822	WSC				
10930	PF00487	FA_desaturase				
**JAA**						
674						
3854	PF00328	His_Phos_2				
3054	PF04479	RTA1				
2694	PF14306,PF01747, PF01583	PUA_2,ATP-sulfurylase,APS_kinase				
3845	PF07350	DUF1479				

Ortho Id	Pfam	Pfam name	CAZymes and AAs[a]	MEROPS[b]	Secondary metab.[c]	Secreted[d]
5270	PF00083	Sugar_tr				
2836	PF00083	Sugar_tr				
5415	PF13520	AA_permease_2				
4766	PF00314	Thaumatin				Y
2401	PF01494	FAD_binding_3				
5537	PF00083	Sugar_tr				
864	PF00248	Aldo_ket_red				
1872	PF07690	MFS_1				
3953	PF07690	MFS_1				
2792	PF02353	CMAS				
4748	PF01124	MAPEG				
5322	PF09994	DUF2235				
1023	PF00295	Glyco_hydro_28				Y
2669	PF01226	Form_Nir_trans				
4650	PF08719	DUF1768				
7879	PF10342	GPI-anchored				
4336						
1473						
6804	PF00128,PF09260	Alpha-amylase,DUF1966	GH13_1			Y
6395	PF01490	Aa_trans				
758	PF00083	Sugar_tr				
3405	PF00106	adh_short				
5305						
5740	PF09286,PF00082	Pro-kuma_activ,Peptidase_S8		S53		Y
9577	PF00732,PF05199	GMC_oxred_N,GMC_oxred_C	AA3_2			Y
2456	PF00004	AAA				
3829						
8640						
9712						
9516	PF00732,PF05199	GMC_oxred_N,GMC_oxred_C	AA3_2			Y
9121	PF00067	p450				
1527	PF13802,PF01055	Gal_mutarotas_2,Glyco_hydro_31	GH31			
9689	PF00248	Aldo_ket_red				
10544	PF07690	MFS_1				
10593	PF00005,PF01061	ABC_tran,ABC2_membrane				
8034						
2008	PF08240,PF13602	ADH_N,ADH_zinc_N_2				
10461						
10493	PF06964	Alpha-L-AF_C	GH51			

Ortho Id	Pfam	Pfam name	CAZymes and AAs[a]	MEROPS[b]	Secondary metab.[c]	Secreted[d]
10494	PF00083	Sugar_tr				
10726	PF00067	p450				
10476	PF03936	Terpene_synth_C				

[a] Gene models of CAZymes (Table S10) and auxiliary redox enzymes (AAs) (Table S8).

[b] Gene models of peptidases (Table S11).

[c] Gene models of natural-product biosynthesis enzymes (Table S13).

[d] Gene models that were predicted to contain a secretion signal using SignalP 4.0.

Table S7 Protein families found among the highly SOM-upregulated orthologues. The table shows the most common Pfam families identified among the 324 orthologue clusters containing genes that were at least 5-fold SOM-upregulated (pairwise comparisons in SOM extract *versus* MMN medium, q<0.01, *n* = 3) (*c.f.* Table S6 and Fig. 3, main manuscript).

Pfam	Description	Species[a]
PF07690	Major Facilitator Superfamily of transporters	7
PF00083	Sugar (and other) transporter	7
PF00248	The aldo-keto reductase family	6
PF01185	Cell wall protein (hydrophobin)	5
PF00067	P450 superfamily of monooxygenases	5
PF00106	The short-chain dehydrogenases/reductases family	5
PF00026	Aspartate proteases	4
PF00704,PF02839	Glycoside hydrolase family 18, carbohydrate-binding module (CBM)	4
PF00011	Heat shock protein (HSP20)	3
PF00732,PF05199	GMC oxidoreductase family	3
PF01425	Amidase	3
PF03169	Oligopeptide transporter protein	3
PF09286,PF00082	Subtilisin-like serine proteases	3
PF13417	Glutathione S-transferases (GSTs)	3
PF00150	Cellulase (glycosyl hydrolase family 5)	3
PF00171	Aldehyde dehydrogenase family	3
PF00491	Arginase family	3
PF03936	Terpene synthase family	3
PF02055	Glycoside hydrolase family 30	3

[a] Number of species containing expressed orthologue(s) with the given Pfam domain.
